# Comparison of time-of-flight and MIEZE neutron spectroscopy of H_2_O

**DOI:** 10.1107/S1600576725003620

**Published:** 2025-06-16

**Authors:** Lukas Beddrich, Johanna K. Jochum, Philipp Bender, Leonie Spitz, Andreas Wendl, Christian Franz, Sebastian Busch, Fanni Juranyi, Christian Pfleiderer, Olaf Soltwedel

**Affiliations:** a Heinz Maier-Leibnitz Zentrum (MLZ), Technische Universität München, D-85748 Garching, Germany; b PSI Center for Neutron and Muon Sciences, CH-5232 Villigen PSI, Switzerland; c Physik Department, Technische Universität München, D-85748 Garching, Germany; d Jülich Centre for Neutron Science JCNS at MLZ, Forschungszentrum Jülich GmbH, D-85748-Garching, Germany; e German Engineering Materials Science Centre (GEMS) at MLZ, Helmholtz-Zentrum Geesthacht GmbH, Garching, Germany; f Munich Center for Quantum Science and Technology (MCQST), Technische Universität München, D-85748 Garching, Germany; g Zentrum für QuantumEngineering (ZQE), Technische Universität München, D-85748 Garching, Germany; h Institut für Physik Kondensierter Materie, Technische Universität Darmstadt, D-64289 Darmstadt, Germany; Universität Duisburg-Essen, Germany

**Keywords:** neutron spin–echo spectroscopy, neutron time-of-flight spectroscopy, modulation of intensity with zero effort, MIEZE, data analysis

## Abstract

We report a comparison of modulation of intensity with zero effort (MIEZE), a neutron spin–echo technique, and neutron time-of-flight (ToF) spectroscopy, a conventional neutron scattering method. The evaluation of the respective recorded signals, which can be described by the intermediate scattering function *I*(*Q*, τ) (MIEZE) and the dynamic structure factor *S*(*Q*, *E*) (ToF), involves a Fourier transformation that requires detailed knowledge of the detector efficiency, instrumental resolution, signal background and range of validity of the spin–echo approximation. It is demonstrated that data obtained from pure water align well within the framework presented here, thereby extending the applicability of the MIEZE technique beyond the spin–echo approximation and emphasizing the complementarity of the two methods. Computational methods, such as molecular dynamics simulations, are highlighted as essential for enhancing the understanding of complex systems. Together, MIEZE and ToF provide a powerful framework for investigating dynamic processes across different time and energy domains, with particular attention required to ensure identical sample geometries for meaningful comparisons.

## Abstract 2

We report a comparison of modulation of intensity with zero effort (MIEZE), a neutron spin–echo technique, and neutron time-of-flight spectroscopy, a conventional neutron scattering method. The basis of this comparison is provided by measurements performed on pure water under the same measurement conditions.

## 1. Introduction

Quasielastic neutron scattering (QENS) describes a limit of inelastic neutron scattering in which the energy transfers are small with respect to the energy of the incident neutron. Three spectroscopic techniques are well established for studies of QENS: time-of-flight (ToF), backscattering (BS) and neutron spin–echo (NSE) spectroscopy. While these methods provide information on correlations in somewhat similar regimes in energy and time as well as momentum and space, the information obtained differs substantially. For instance, BS and ToF spectroscopies determine the change in energy of the neutron by means of a crystal analyser or the time of flight of the neutron, respectively, whereas NSE exploits the precession of the neutron spin in a suitable magnetic field. Further, BS and ToF provide the dynamic structure factor *S*(*Q*, *E*), while NSE provides the intermediate scattering function *I*(*Q*, τ), where *Q*, *E* and τ are the momentum transfer, energy transfer and spin–echo (SE) time, respectively. All methods have their strengths and weaknesses, yet conceptual differences in the data collected and the scientific insights gained have been discussed controversially in the scientific community.

The aim of this paper is to highlight the complementarity of NSE and ToF and to illustrate key aspects when comparing data. As our main message, we argue that an unambiguous understanding of complex physical processes, which are forcibly addressed in cutting-edge research, requires the combination of both *S*(*Q*, *E*) and *I*(*Q*, τ).

Neutron ToF spectroscopy infers the kinetic energy of a neutron from the time of flight between two known points in space. In indirect-geometry ToF instruments, the sample is illuminated by a pulsed white beam and the energy of the scattered beam is determined using a crystal analyser. In contrast, in direct-geometry ToF instruments the incident beam is monochromated either by a crystal monochromator or by a set of at least two choppers. In both cases the final energy is inferred from the time of flight in traversing the distance between the sample and the detector.

ToF spectrometers have the advantage of large detector coverage. However, at neutron sources such as reactors which provide a continuous neutron flux, they suffer from a dramatic reduction in flux due to the chopping of the beam. In contrast, for pulsed beams provided by spallation sources, ToF instruments appear to be a natural choice. ToF instruments are quite versatile, with an energy resolution as high as a few microelectronvolts. Typical instruments cover a large range of momentum transfers *Q* that encompass atomic and inter­atomic distances below 10 Å but lack resolution on mesoscopic length scales above 10 Å due to their relaxed beam collimation.

Important examples include LET at ISIS (Didcot, UK) (Bewley *et al.*, 2011 ▸; Nilsen *et al.*, 2017 ▸) or IN5 at the ILL (Grenoble, France) (Ollivier *et al.*, 2002 ▸; Ollivier & Mutka, 2011 ▸). As another advantage, ToF spectrometers may use incident energies of up to 1000 meV, such as MARI (Andersen, 1996 ▸; Le *et al.*, 2023 ▸). In addition, the use of polarized beams (Zaliznyak *et al.*, 2005 ▸) and polarization analysis are available (Winn *et al.*, 2015 ▸; Bewley *et al.*, 2011 ▸).

Neutron BS spectroscopy utilizes crystals in (nearly) perfect backscattering geometry to analyse the energy of the scattered neutrons. The energy of the incoming neutron beam is varied using a Doppler monochromator. This configuration offers high energy resolution with a limited dynamic range, and instruments at reactor sources offer the highest energy resolution, with a maximum of 0.3 eV on IN16B at the ILL (Gardner *et al.*, 2020 ▸; Frick *et al.*, 2010 ▸). In comparison, instruments at spallation sources offer a broader dynamic range at a somewhat reduced resolution. The largest dynamic range of ±3.5 × 10^3^ µeV can be reached on the spectrometer IRIS at ISIS (Demmel *et al.*, 2018 ▸). Analogously to ToF, BS spectrometers include a large detector bank, offering simultaneous information on a large range in *Q* space. Instruments with an Si(111) analyser crystal typically reach up to *Q* = 1.8 Å^−1^ at the highest energy resolutions, while higher *Q* of up to 3.8 Å^−1^ may be reached at a reduced energy resolution, using a graphite or Si(311) analyser (Gardner *et al.*, 2020 ▸).

Taken together, ToF and BS are widely used to study *e.g.* molecular reorientation, hydrogen diffusion and liquid dynamics. However, ToF and BS cannot resolve the dynamics on mesoscopic length scales, such as domain motion in macromolecules, polymer chain dynamics or emergent excitations in quantum magnets. This shortcoming is due to the combination of intermediate energy resolution and relatively poor resolution of momentum transfer at small *Q*.

NSE techniques are well known for achieving very high energy resolutions down to below 1 neV (Holderer & Ivanova, 2015 ▸; Farago *et al.*, 2015 ▸). By decoupling the energy resolution from the wavelength spread, a very high neutron intensity is reached (Mezei, 1972 ▸; Mezei, 1980 ▸). In NSE, comparison of the total phase of the Larmor precession of the neutron spin, acquired in a well defined magnetic field region before and after the sample, serves to encode energy transfers due to scattering. NSE is especially well established in the investigation of slow relaxation processes of the order of ∼1 to ∼100 ns. Typical scientific problems addressed with NSE are thermal fluctuations of surfactant membranes in microemulsions (Mihailescu *et al.*, 2001 ▸), the molecular rheology of polymer melts (Schleger *et al.*, 1998 ▸), dynamics in lipid phases (Nylander *et al.*, 2017 ▸), thermally activated domain motion in proteins (Bu *et al.*, 2005 ▸), relaxation phenomena in networks and rubbers (Salatto *et al.*, 2021 ▸), interface fluctuations in complex fluids like emulsions (Kyrey *et al.*, 2019 ▸) and polyelectrolytes (Kanaya *et al.*, 1989 ▸), transport processes in polymeric electrolytes (Hopfenmüller *et al.*, 2018 ▸), and domain dynamics of proteins (Biehl & Richter, 2014 ▸) or enzymes (Inoue *et al.*, 2010 ▸). Typically, an NSE measurement only covers a small part of momentum space compared with ToF spectroscopy. However, wide-angle NSE variants have been developed such as SPAN (Pappas *et al.*, 2000 ▸) and WASP (Fouquet *et al.*, 2007 ▸).

To increase the resolution of NSE, the field integral seen by the neutron has to be increased. The associated technical challenges regarding the field homogeneity have been addressed in different ways. Most importantly, highly sophisticated correction coils have been developed (Monkenbusch, 1990 ▸). However, this approach is limited by the energy density stored in these coils and the mechanical forces generated. To overcome these limitations of classical NSE instruments, superconducting coils have been developed (Pasini *et al.*, 2019 ▸; Walter *et al.*, 2009 ▸), offering increased field homogeneity and higher magnetic fields.

Pursuing a different approach to overcome the limitations of classical NSE, Golub & Gähler (1987 ▸) proposed the resonant neutron spin–echo technique (NRSE), where the solenoids are replaced by a pair of radio-frequency (RF) neutron spin-flippers. In NRSE, different SE times are reached by tuning the RF flippers to different frequencies.

While classical NSE and NRSE are well established in studies of soft matter, several shortcomings are known in studies of hard condensed matter. Perhaps most importantly, a reduction or total loss of signal occurs in depolarizing samples or when using depolarizing sample environments. Measurements under such depolarizing conditions are cumbersome, *e.g.* as witnessed for ferromagnetic NSE (Mezei *et al.*, 2001 ▸; Keller *et al.*, 2022 ▸). Further, signal contributions due to in­coherent scattering may reduce signal contrast substantially. In addition, studies in the limit of small momentum transfer *Q* will suffer from substantial background scattering.

To overcome these limitations of NSE and NRSE, modulation of intensity with zero effort (MIEZE) may be used, representing a variant of NRSE. Analogously to NRSE (Golub & Gähler, 1987 ▸; Gähler *et al.*, 1992 ▸), MIEZE uses RF spin-flippers, instead of large solenoids, to create a precession zone for the neutron spin. However, in MIEZE the pair of RF spin-flippers before the sample is operated at different frequencies, leading to an intensity modulation of the signal behind the analyser with a frequency *f*_M_ = 2(*f*_*B*_ − *f*_*A*_). If the analyser is placed in front of the sample, depolarizing effects by the sample or sample environment no longer affect the measurements. Moreover, unlike the polarization in conventional NSE, the MIEZE contrast is not reduced by incoherent spin-flip scattering in the sample (Gähler *et al.*, 1992 ▸).

Since the RF flippers are compact, they allow for the insertion of a field-subtraction coil between them (Jochum *et al.*, 2019 ▸; Häussler & Schmidt, 2005 ▸). This permits extension of the dynamic range of MIEZE and NRSE by several orders of magnitude towards shorter echo times, deep into the nominal range of energy transfers covered by ToF or BS. For instance, on the RESEDA instrument SE times as short as τ_min_ = 0.1 fs may be reached at a wavelength of 4.5 Å (Jochum *et al.*, 2022 ▸). However, this advancement towards shorter SE times, and therefore putatively larger energy transfers, reaches well beyond the SE approximation representing the standard framework underlying NSE in the limit of small energy transfers (Franz *et al.*, 2019*a* ▸; Franz *et al.*, 2019*c* ▸).

In this paper we consider the validity of NSE techniques in parameter regimes at the border of and outside the SE approximation. In principle, MIEZE investigations are limited to dispersionless excitations, unless additional sample information, such as molecular dynamics (MD) simulations or input from other neutron spectroscopic methods, is available (Keller *et al.*, 2022 ▸). However, a significant advantage is that MD simulations can generate intermediate scattering functions using force field parameters – now increasingly refined with machine learning (Unke *et al.*, 2021 ▸) – which, when processed with the transformation algorithm presented here, can be accurately converted into MIEZE spectra, enabling the optimization of these parameters. The algorithm presented here essentially follows the van Hove formalism, with a focus on instrument-specific resolution effects in both reciprocal and real space, as well as in energy and frequency space.

## 2. Theoretical framework

In the following, we present the theoretical framework for NSE and the MIEZE technique, as some of the concepts discussed have a significant impact on the data presented below. For a theoretical background on neutron ToF spectroscopy, we refer the reader to standard works such as Bee (2025 ▸). NSE techniques are based on the precession of the neutron spin in magnetic fields as a probe that allows the inference of energy transfers during scattering events. For an introduction we refer to the book by Mezei *et al.* (2003 ▸). In the following we focus on the concepts needed to discuss the SE approximation and its implications. We start by defining the precession angle of a neutron travelling with a velocity *v* perpendicular to a magnetic field of field strength *B* and length *L*:
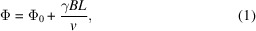
where γ is the neutron’s gyromagnetic ratio (γ = 183.25 MHz T^−1^ for angles in radians). Without loss of generality, we assume Φ_0_ = 0 in the following. In an NSE setup, a neutron travels across a well defined magnetic field region (*B*_1_, *L*_1_) before reaching the sample, followed by a trajectory across a second well defined field region after the sample position (*B*_2_, *L*_2_). The phase Φ_D_ of the neutron at the detector position may be written as
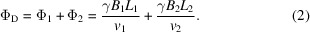
Choosing the lengths and field strengths such that *L*_2_ = *L*_1_ = *L* and *B*_2_ = − *B*_1_ = *B*, equation (2) becomes 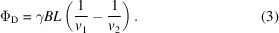
Writing *v*_2_ = *v*_1_ + Δ*v*, where Δ*v* is the change in velocity the neutron undergoes when interacting with a sample positioned between the precession fields, one obtains
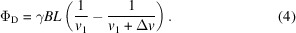
For a purely elastic scattering process, Δ*v* = 0 and therefore Φ_D_ = 0. If the neutron exchanges energy with the sample, Δ*v* ≠ 0, resulting in a phase shift Φ_D_. The change in neutron energy during such an interaction may be written as
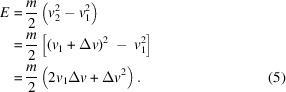
The SE approximation assumes 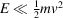, *i.e.* the energy transfer *E* is much smaller than the kinetic energy 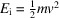 of the incoming neutrons. Thus 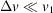, from which it follows that
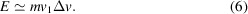
In turn, Δ*v* can be written as
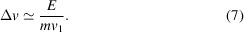
Within the SE approximation Δ*v* + *v*_1_ ≃ *v*_1_ and therefore
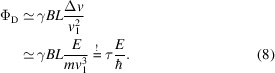
Assuming the SE approximation, equation (8) hence defines the SE time τ as a proportionality factor between the neutron phase Φ_D_ at the detector and the energy transfer *E*.

Using a neutron polarization analyser and a neutron detector at a certain scattering angle 2ϑ, the number of polarized neutrons is recorded. This corresponds to the expectation value of 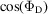 over the scattered neutrons (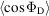) in 2ϑ. Within the SE approximation, 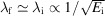, the momentum transfer *Q* is well defined via 2ϑ and the probability of a scattering event with energy transfer *E* is given by *S*(*Q*, *E*). This assumption leads to
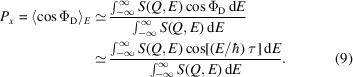
The numerator of equation (9) is the cosine Fourier transform of *S*(*Q*, *E*), which represents the real part of the time-dependent correlation function, also known as the intermediate scattering function *I*(*Q*, τ). The denominator is the static structure factor *S*(*Q*). Therefore,
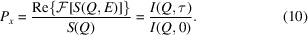
Within the SE approximation, this leads to the interpretation that the measured polarization *P*_*x*_ is essentially the energy cosine transform of *S*(*Q*, *E*) normalized by the static structure factor. Alternatively, expressed in the time domain this represents the intermediate scattering function normalized to its value at zero Fourier time.

### 2.1. NSE beyond the SE approximation

**Figure 1:**
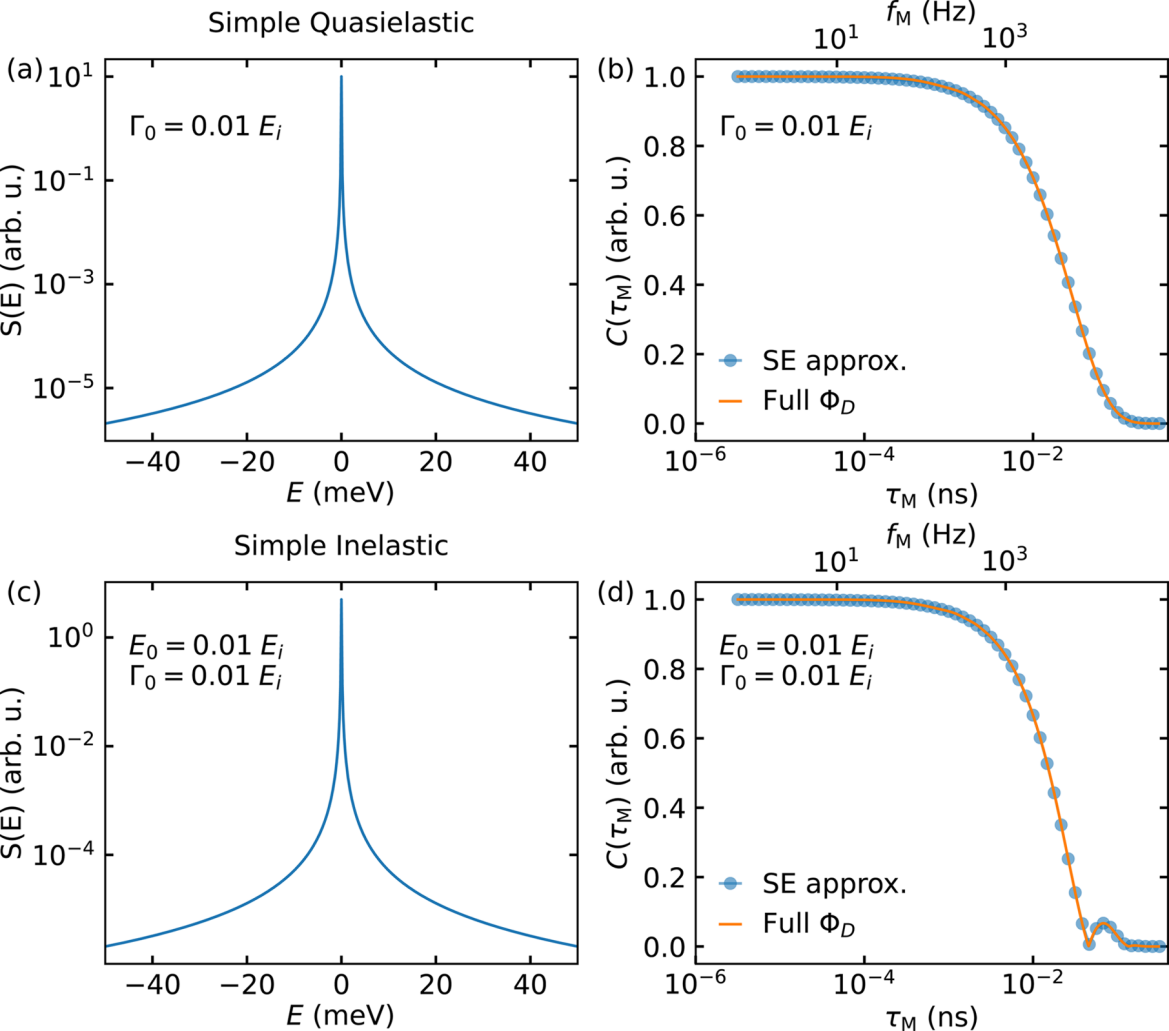
Illustration of typical signals observed in MIEZE spectroscopy. The wavelength of the incoming neutron beam was assumed to be monochromatic and λ = 6.0 Å, which corresponds to *E*_i_ = 2.27 meV. (*a*) The dynamic structure factor described by a Lorentzian with a narrow linewidth Γ_0_ = 0.01*E*_i_, corresponding to quasielastic scattering. (*b*) The curve shown in panel (*a*) after the application of equation (9) (SE approximation, blue circles) and equation (21) (explicit calculation, solid orange line). (*c*) The dynamic structure factor described by a Lorentzian with a narrow linewidth Γ_0_ = 0.01*E*_i_ and a small energy transfer *E*_0_ = 0.01*E*_i_, corresponding to low-energy inelastic scattering. (*d*) The curve shown in panel (*c*) after the application of equation (9) (SE approximation, blue circles) and equation (21) (explicit calculation, solid orange line).

Assuming a typical wavelength of λ = 6 Å for the incoming neutrons, corresponding to a kinetic energy of *E*_i_ = 2.27 meV, the SE approximation holds true for quasielastic scattering processes with energies in the microelectronvolt range (Fig. 1). Accordingly, NSE as it was originally conceived is not suited to investigating processes with energy transfers in the milli­electron­volt range. However, as we will show below, using for example shorter wavelengths or state-of-the-art numerical methods, SE techniques can be pushed beyond their original parameter space.

The first subtle yet important difference that arises when considering larger energy transfers is the necessity of accounting for the wavevector transfer. In its general form it reads 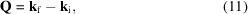
with its magnitude given by the law of cosine,
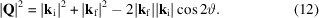
Within the SE approximation, the energy transfer remains small (less than 10% of the incoming neutron energy), ensuring that |**k**_f_| ≃ |**k**_i_| and correspondingly λ_f_ ≃ λ_i_. This significantly simplifies the expression for the momentum transfer *Q*, relating it directly to the neutron wavelength and scattering angle through the well known equation
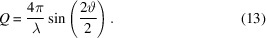
Strictly speaking, the measured quantity given in equation (10) now explicitly depends on the scattering angle rather than the wavevector transfer, which remains undefined, as all energy transfers are inherently permitted.

The second important difference is the probability of a scattering event which, in the more general case, is described by the double differential cross section and is related to the dynamic structure factor by
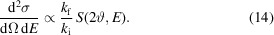
Note that the structure factor is defined here as a function of the scattering angle, which is necessary for comparing SE and ToF spectroscopy techniques. If *k*_f_/*k*_i_ ≃ 1, as assumed in the SE approximation, it is sufficient to consider only the dynamic structure factor *S*(*Q*, *E*) instead of the double differential cross section (Zolnierczuk *et al.*, 2019 ▸).

The third important factor to consider is the wavelength-dependent resolution function of an NSE instrument. Typically, *I*(*Q*, τ) is normalized not to the full integral 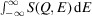 but to a structure factor where the integral is taken only over the band pass of the spectrometer (Richter *et al.*, 1998 ▸). The lower boundary of this integral is given by −*E*_i_, the energy of the incoming neutron, and the upper boundary is given by the maximum wavelength accepted by the neutron polarization analyser. The integral is further weighted by the transmission functions of the materials in the beam path and the detector efficiency.

Finally, the explicit expression for the phase at the detector Φ_D_ must be taken into account [see equation (4)].

Taken together, equation (9) then becomes 
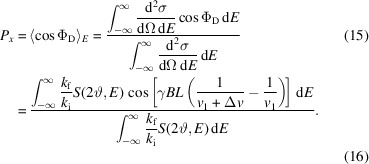
Here, 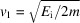, with the incoming neutron energy *E*_i_, and Δ*v* is as defined above.

### 2.2. The MIEZE method

The energy-dependent transmission function of an SE instrument is non-trivial to describe as it involves a variety of elements, including wavelength-dependent spin flip, spin analysis, magnetic guide fields, adiabatic transition fields and absorption. In contrast, this is not the case for MIEZE, as all spin manipulation is performed on the unperturbed beam prior to the sample.

The data presented below were recorded using the MIEZE technique (Gähler *et al.*, 1992 ▸). In a MIEZE setup a pair of resonant spin flippers before the sample are operated at different frequencies, *f*_*A*_ and *f*_*B*_, and separated by a distance *L*_*AB*_ (Golub & Gähler, 1987 ▸). Similarly to classical NSE, the spin phase at the detector may be defined as (Golub *et al.*, 1994 ▸; Keller *et al.*, 2002 ▸)
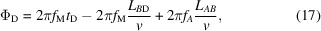
where *t*_D_ represents the time of flight of the neutron from the first spin flipper to the detector, *v* is the initial neutron velocity before the sample, *f*_M_ = 2(*f*_*B*_ − *f*_*A*_) is the MIEZE frequency and *L*_*B*D_ is the distance between the second spin flipper and the detector. The MIEZE detector is placed such that the velocity-dependent terms cancel out, referred to as the MIEZE condition, simplifying the equation (Gähler *et al.*, 1992 ▸; Jochum *et al.*, 2019 ▸).

Energy transfers *E* during the scattering event will induce a delay Δ*t*_D_ in the neutron flight time over the distance between the sample and the detector *L*_SD_:
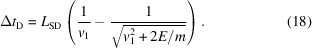
This leads to a change in the spin phase at the detector by
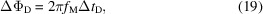
which reduces the contrast of the time-dependent intensity variation.

Within the SE approximation, this equation may be written by exact analogy to classical NSE spectroscopy as
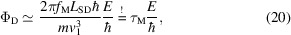
where the Fourier time τ_M_, also known as the MIEZE time, represents a proportionality factor between the phase at the detector and the energy transfer.

Averaging this effect over all possible energy transfers, by analogy to the polarization *P*_*x*_ for classical NSE [see equation (15)], results in the following general expression for the contrast:
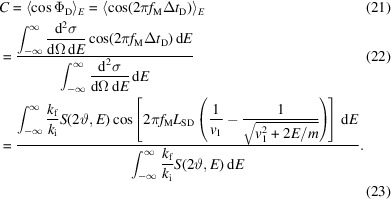


### 2.3. Computation of the MIEZE contrast

To elucidate the physical meaning of the MIEZE contrast *C*, it is instructive to discuss a few illustrative examples. To evaluate the importance of the SE approximation, the expected MIEZE contrast was computed for different dynamic structure factors *S*(*Q*, *E*), assuming a monochromatic neutron beam with a wavelength λ = 6 Å, corresponding to a kinetic energy of *E*_i_ = 2.27 meV.

Two simple examples for quasi- and inelastic scattering are first considered, as shown in Fig. 1 ▸. For the purposes of these simple examples, the following definitions of quasi- and inelastic scattering are adopted. Quasielastic scattering is considered as the limiting case of inelastic scattering, where the spectrum is defined by a central peak at *E* = 0, corresponding to a broadening of the elastic line. In the cases presented here, the energy transfers are only 1% of the incoming neutron energy *E*_i_, indicating a dynamic structure factor for which the SE approximation remains valid. As a result, applying equations (9) and (21) to *S*(*Q*, *E*) produces the same curve. A simple quasielastic signal is represented in *S*(*Q*, *E*) as *S*(*E*) ∝ 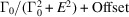, where Γ_0_ is the linewidth and Offset denotes a time-independent background [Fig. 1 ▸(*a*)]. This results in an exponentially decaying signal 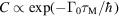 in the time domain, from which a characteristic timescale τ_R_ can be extracted [Fig. 1 ▸(*b*)]. This characteristic timescale is connected to the linewidth (HWHM) of the Lorentzian in *S*(*Q*, *E*) via τ_R_ = ℏ/Γ_0_.

A corresponding inelastic signal, described as *S*(*E*) ∝ 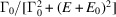 + 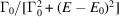 + Offset, where Γ_0_ and Offset are defined as above and *E*_0_ denotes the finite energy transfer, is shown in Fig. 1 ▸(*c*). For ease of comparison with the quasielastic case, Lorentzians are also used to describe the inelastic case. Applying equations (9) and (21) to this Lorentzian leads to curves that comprise an exponential decay with *C* ∝ 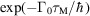 modulated by a 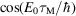 term, as shown in Fig. 1 ▸(*d*). Next, these simple examples are modified to emphasize the influence of larger energy transfers and a time-independent background.

**Figure 2:**
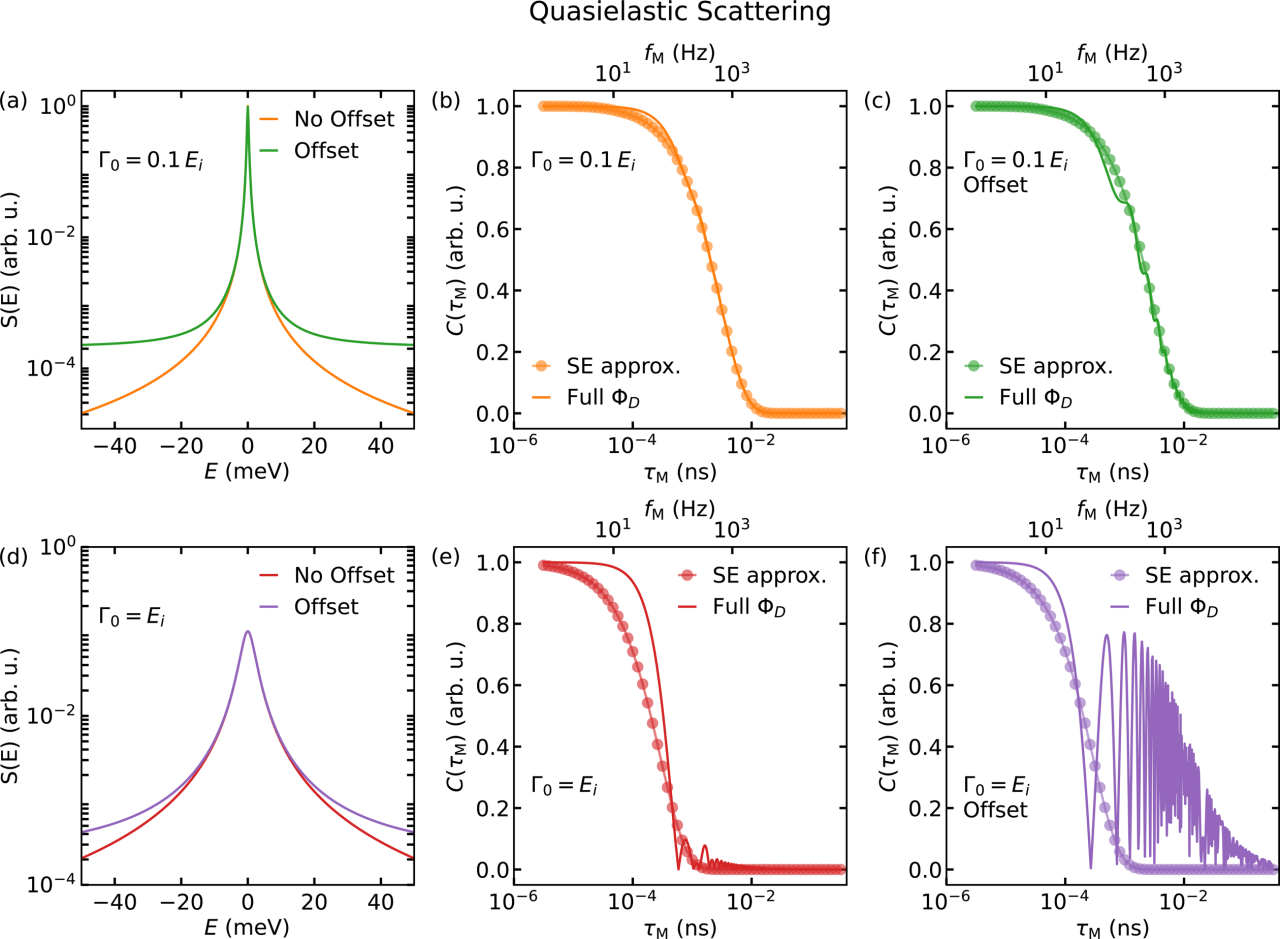
Illustration of the difference between the SE approximation and the explicit phase calculation for quasielastic scattering. Panels (*a*) and (*d*) show two dynamic structure factors whose transformation into the time domain is shown in graphs (*b*) and (*c*), and (*e*) and (*f*), respectively, as indicated by their colour. The transformation calculated by the SE approximation is shown as circles, while the solid lines depict the result of equation (21). The wavelength of the incoming neutron beam was assumed to be monochromatic and λ = 6.0 Å, which corresponds to *E*_i_ = 2.27 meV.

Four Lorentzian distributions describing the quasielastic case are shown in Fig. 2 ▸. Application of equation (9) to the Lorentzians in Fig. 2 ▸(*a*) produces the curves shown by circles in corresponding colours in Figs. 2 ▸(*b*) and 2 ▸(*c*). Using equation (21) – applicable independently of the measured excitation energy – to calculate the contrast of the curves in Fig. 2 ▸(*a*) yields the continuous lines, also colour-matched. For a linewidth Γ_0_ = 0.1*E*_i_, small deviations between the two curves occur [Fig. 2 ▸(*b*)]. Adding a constant background to the Lorentzian with Γ_0_ = 0.1*E*_i_ results in the green curve in Fig. 2 ▸(*a*) and leads to an oscillatory modulation on top of the exponential decay, as shown in Fig. 2 ▸(*c*). When increasing the linewidth further to Γ_0_ = *E*_i_, significant discrepancies are observed between the SE approximation and the explicit calculation [Fig. 2 ▸(*e*)]. Analogously to the case of Γ_0_ = 0.1*E*_i_, the addition of a constant background leads to an oscillatory modulation atop the exponential decay [Fig. 2 ▸(*f*)]. These oscillations, which arise for large Γ_0_ or increased background, are due to the nature of the finite integration window of the Fourier transform (Wuttke *et al.*, 1995 ▸). In the first approximation the finite energy window, bounded below and above by the maximum neutron energy loss and the highest detectable neutron energy, respectively, may be approximated by a top-hat function. Therefore, effectively a Fourier transform of *S*(2ϑ, *E*) convoluted with a top-hat function is performed. As the Fourier transform of a top-hat function is a sinc function, this results in a contrast *C* modulated with a sinc function, *i.e.* a damped oscillation along the positive *x* axis, with an amplitude that decreases as 1/*n*. Here, *n* is defined via the Taylor series sinc(*x*) = 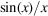 = 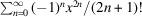. These oscillations are absent from the SE approximation [Figs. 2 ▸(*c*) and 2 ▸(*f*)]. Consequently, oscillations indicative of purely inelastic scattering in the SE approximation may also be present when measuring a quasielastic scatterer. These two contributions cannot easily be disentangled, noting that the parameters used for this illustration in Figs. 2 ▸(*e*) and 2 ▸(*f*) are somewhat unrealistic and chosen to highlight the basic mechanism.

**Figure 3:**
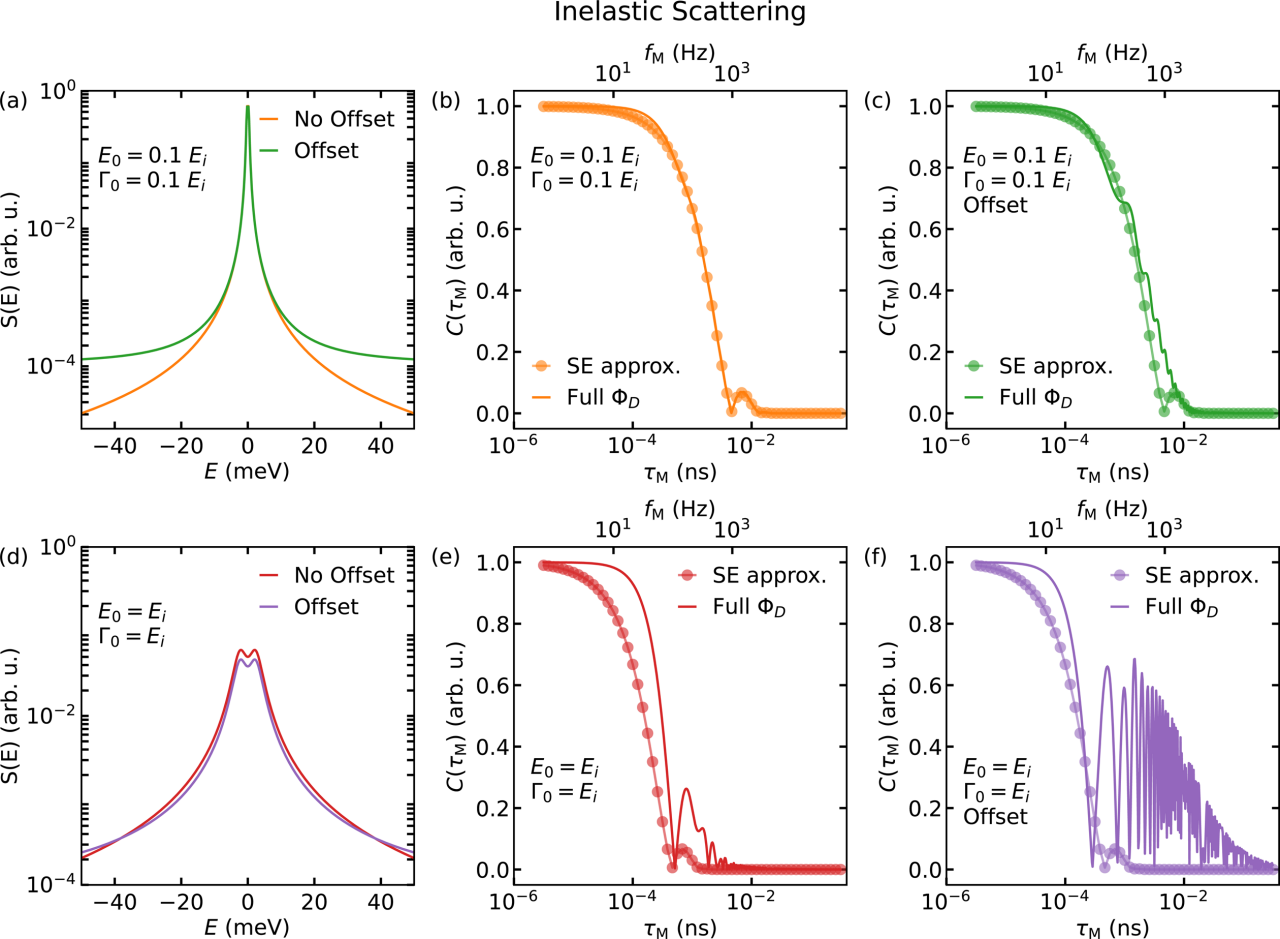
Illustration of the difference between the SE approximation and the explicit phase calculation for inelastic scattering. Panels (*a*) and (*d*) show two dynamic structure factors whose transformation into the time domain is shown in graphs (*b*) and (*c*), and (*e*) and (*f*), respectively, as indicated by their colour. The transformation calculated by the SE approximation is shown as circles, while the solid lines depict the result of equation (21). The wavelength of the incoming neutron beam was assumed to be monochromatic and λ = 6.0 Å, which corresponds to *E*_i_ = 2.27 meV.

Fig. 3 ▸ visualizes, in the same layout as Fig. 2 ▸, true inelastic scattering. Fig. 3 ▸(*a*) shows two Lorentzians for finite excitation energies *E*_0_. Similarly to the quasielastic case, the SE approximation and the explicit calculation [equation (21)] agree well if *E*_0_ and Γ_0_ are smaller than 10% of *E*_i_. For increasing Γ_0_ and *E*_0_ the deviations increase as well, leading first to a mismatch in the curvature of the decay and then to additional oscillations for equation (21). Analogously to the quasielastic case, we can see that an increased background leads to additional oscillations.

The features discussed here, though presented as examples, are observed in real data. They must be taken into account when analysing MIEZE and classical NSE data, as well as when applying a Fourier transform to ToF data to separate contributions to the resolution function (Zorn, 2012 ▸).

## 3. Experimental methods and data reduction

Three neutron scattering measurements on liquid Milli-Q ultrapure water were performed. Firstly, MIEZE measurements were conducted on the resonant SE spectrometer RESEDA (Franz & Schröder, 2015 ▸; Franz *et al.*, 2019*b* ▸; Franz *et al.*, 2019*a* ▸) at the MLZ. The measurements were performed under a scattering angle 2ϑ = 22° using a wavelength λ = 6 Å. For the measurements, the water was kept in a Hellma macro-cell with an optical path length of 1 mm. The cell thickness was chosen to optimize the scattering signal. Previous measurements using a Hellma macro-cell with an optical path length of 0.2 mm suffered from low statistics. However, in terms of evaluated translational diffusion coefficients, the data extracted from the measurements using the different cells agreed well with each other and with literature values. The cell was kept at a temperature of 300 K using a closed-cycle cryostat in closed-loop temperature control. The resolution curve was recorded using carbon powder, representing a purely elastic scatterer, inside an identical Hellma macro-cell. The data were reduced using the software package *MIEZEPY* (Schober *et al.*, 2019 ▸). This procedure includes the normalization of the sample contrast to the contrast of a purely elastic scatterer to take into account all instrument-specific resolution effects, such as the different wavelengths and wavelength bands used, potential polarization losses in the primary spectrometer, scattering off the sample environment, and intrinsic background scattering off instrument components. The process yields the contrast *C*(2ϑ, *f*_M_) at a fixed scattering angle 2ϑ.

ToF measurements were performed on the spectrometers FOCUS at SINQ (Janßen *et al.*, 1997 ▸; Mesot *et al.*, 1996 ▸) and TOFTOF at the MLZ (Unruh *et al.*, 2007 ▸) using a wavelength λ = 6 Å. For both measurements the sample cell was kept at a temperature of 300 K using a closed-cycle cryostat in closed-loop temperature control. For the measurements performed on FOCUS the water was kept in an identical sample cell to that used for the RESEDA measurements, while for the measurements performed on TOFTOF a hollow cylindrical aluminium container with outer diameter *d*_1_ = 22.5 mm and inner diameter *d*_2_ = 22.2 mm, and therefore a path length of 0.15 mm, was used. The influence of the difference in sample thickness on the comparability of MIEZE and ToF data is discussed below. For the data acquired on FOCUS, the reduction was performed using the *Data Analysis and Visual­ization Environment* (*DAVE*) (Azuah *et al.*, 2009 ▸), while TOFTOF data were treated with *Mantid* (Arnold *et al.*, 2014 ▸). For both data sets the same procedure was applied. The data were first normalized to the incoming flux to account for small fluctuations. To account for scattering off the cuvette, an empty cell was measured and the data subsequently subtracted. The detector was calibrated using a vanadium standard, taking into account a correction for the anisotropy of the Debye–Waller factor of vanadium. The vanadium measurement was also used to determine the resolution of the elastic line. Subsequent to these corrections, the energy transfer was calculated from the measured neutron time of flight.

Knowing the energy transfer and the scattering angle 2ϑ, the momentum transfer *Q* may be calculated. However, this step was omitted since a comparison with the data recorded on RESEDA was only possible for constant scattering angle 2ϑ. As a final step, the measured neutron intensity was corrected for the energy-dependent detector efficiency (Unruh *et al.*, 2007 ▸) and for the *k*_f_/*k*_i_ factor in the double differential cross section.

Before discussing the neutron scattering data recorded on ultra-pure water, it is helpful first to examine the vanadium resolution data measured on TOFTOF in the light of the observations presented in Section 2.3.

**Figure 4:**
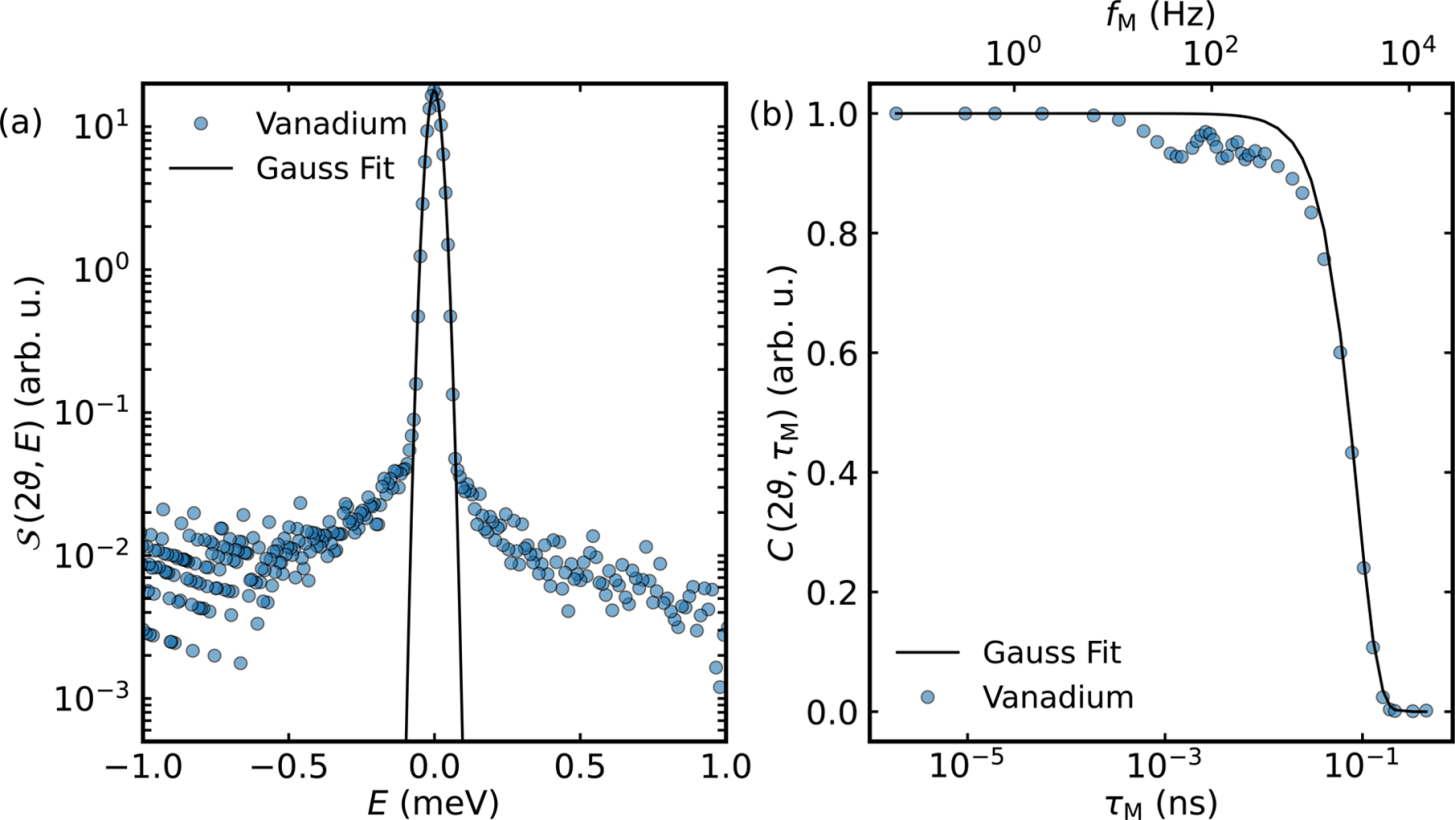
Vanadium data recorded on TOFTOF. (*a*) Vanadium data in energy space, shown together with a Gaussian fit to the data. (*b*) The data set and fit shown in panel (*a*) after the MIEZE transform [equation (21)] is applied.

As can be observed in Fig. 4 ▸, application of equation (21) to the vanadium data and to a Gaussian fit to these data yields differing results. The vanadium sample was measured as a powder in the same sample container as the water sample, with the primary difference being a path length of 2 mm for the vanadium to increase scattering. The MIEZE transform applied to the Gaussian leads to a smoothly decaying curve, while the MIEZE transform of the data themselves exhibits oscillations. These oscillations originate from the time-independent instrumental background, which stretches over a wide range in energy up to the cutoff for the integration window used during the transform.

## 4. Data analysis

Two separate approaches were taken to compare the data measured at the three different spectrometers: (i) a direct approach, namely a transformation between time and energy domains, and (ii) an indirect approach, namely a parallel fit of model functions. Both data evaluation pathways are described in the following.

### 4.1. Direct approach: transformation between time and energy domains

**Figure 5:**
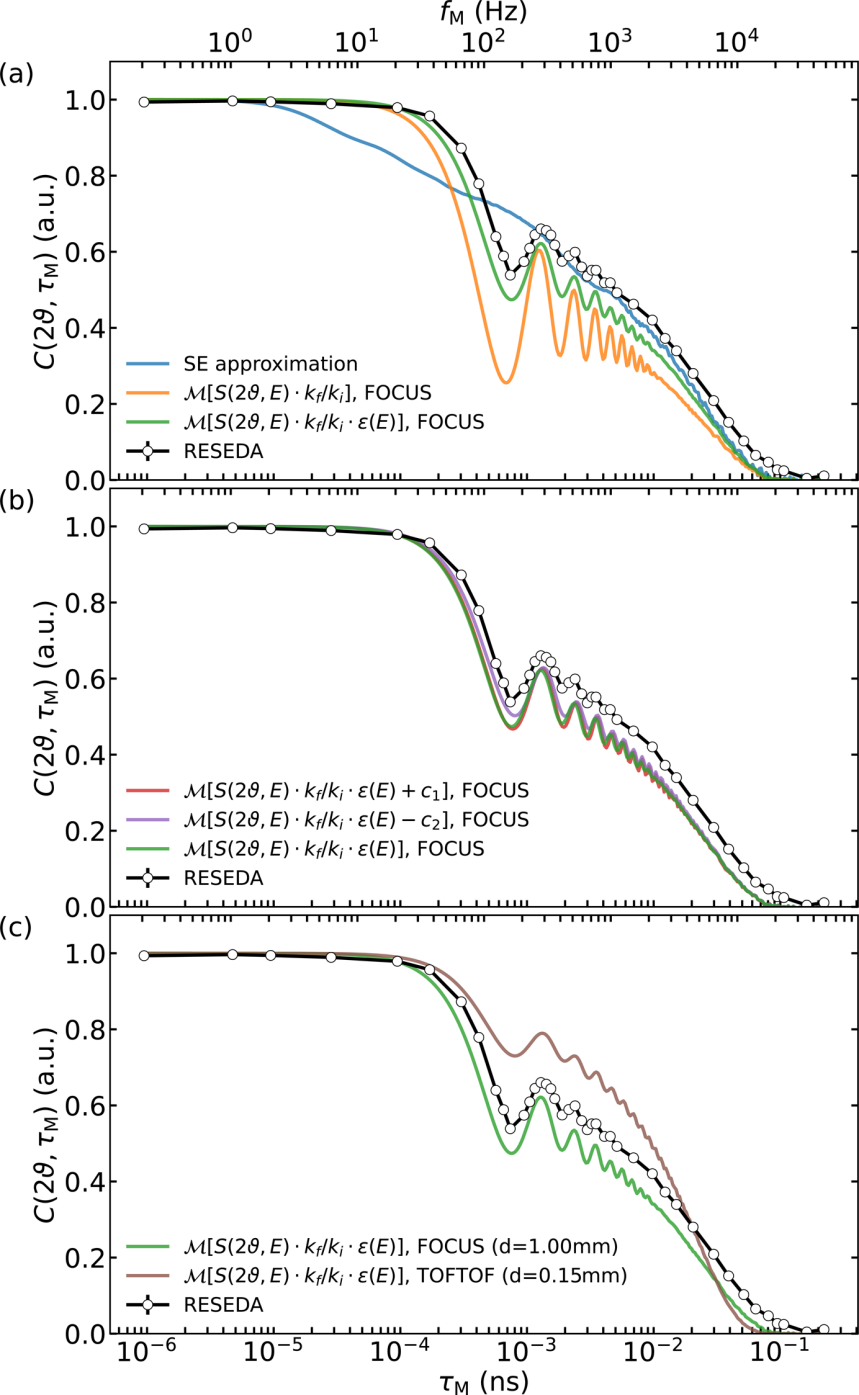
Direct transformation of the recorded ToF spectra *S*(2ϑ = 22°, *E*) into the time domain. (*a*) The transformed ToF spectrum of FOCUS with increasing number of corrections for direct comparison with the RESEDA data (circles). The simplest case uses the SE approximation [equation (9)] and results in the blue curve. The orange curve is obtained by transforming the energy spectrum with equation (21), which reproduces quite accurately the frequency of the oscillations visible in the RESEDA data. The green line includes the energy-dependent detection efficiency of the CASCADE detector, which now gives the correct amplitude of the oscillations. Comparing this last with the MIEZE data, at high frequencies the transformed ToF data decay faster and at medium frequencies the contrast is about 0.05 lower than expected. (*b*) A visual­ization of the effect of adding (red line) or subtracting (purple line) a constant background to the FOCUS data before applying the trans­formation, leading to a downward or upward shift compared with the original curve, respectively. (*c*) A comparison of the RESEDA data (circles) with the Fourier transforms of the different ToF data sets. The data measured with the same sample cell as RESEDA (green line, measured on FOCUS) match the RESEDA data much better than the data recorded in a much thinner hollow cylinder (brown line, measured on TOFTOF), emphasizing the impact of sample thickness and multiple scattering.

As outlined in Sections 2.2 and 2.3, it proves to be essential to go beyond the SE approximation to transform the ToF energy spectrum. The results of applying equations (9) and (21) to the data measured on the flat Hellma cuvette (FOCUS) and the hollow cylinder (TOFTOF) compared with the data measured on RESEDA (circles) are depicted in Fig. 5 ▸. The error bars representing the statistical uncertainties in the contrast are smaller than the markers themselves. In Fig. 5 ▸(*a*) an increasing number of corrections to the simple SE approximation were taken into account in the transformation of the FOCUS data. Emphasizing the inadequacy of the SE approximation, the blue line was calculated using the standard framework expressed by equation (9), which is unable to describe the MIEZE data. Adding the full expression of the MIEZE phase change ΔΦ_D_ and the correction factor *k*_f_/*k*_i_, introducing a natural cutoff at *k*_f_ = 0, yields the orange curve, which reproduces the characteristic oscillations in terms of their frequency but not with the correct amplitude. Factoring in the energy-dependent detection efficiency of the CASCADE detector on RESEDA (Köhli *et al.*, 2016 ▸), designated as ɛ(*E*), achieves a reduction in the amplitude of the oscillations around 100 to 1000 Hz to the exact level seen in the MIEZE data (green curve). This transformation, including the full expression of the MIEZE phase, the factor *k*_f_/*k*_i_ and the CASCADE detector efficiency, is referred to as ‘MIEZE transformation’ in the following.

Despite these refinements, it remains impossible to reproduce the exact numerical values of the MIEZE data, in either the mid-frequency or high-frequency ranges. These discrepancies have distinct origins, which we now describe.

For the mid-frequency range, the main contribution to the difference between the RESEDA data and the transformed ToF data appears to be due to the background of the energy spectrum and its treatment. This can be seen in Fig. 5 ▸(*b*), where the MIEZE transformation of the FOCUS data (green line) is compared with the same data set modified by adding a constant background *c*_1_ = 0.058 or by subtracting *c*_2_ = 0.035. This seems minuscule compared with the peak value of the measured intensity on FOCUS, *S*(2ϑ, *E*)_max_ = 1593 (arbitrary units). However, the resulting contrast curve has been noticeably shifted upwards (purple line) and downwards (red line) in the mid- to low-frequency ranges, respectively, while the general structure of the oscillations has been preserved. When adding a constant background, the contrast shifts down, because in relative terms more integrated intensity is added to the high-energy part of the spectrum. Integrated over the entire energy range of *S*(2ϑ, *E*), the contributions of the constants *c*_1_ and *c*_2_ account for about 2.2% and 1.3% of the spectrum, respectively. This shows that, for a successful and quantitative comparison with MIEZE data, a detailed knowledge of the instrument background, especially at high energies, is essential. Spurious inelastic scattering at such high energy transfers cannot be entirely ruled out, but it appears unlikely to be the cause of the additional background. The dark count rate of the ToF detectors on the other hand could prove to be crucial. Dark counts are evenly distributed across the time bins and manifest as a constant background along the resolved energy band. These finite values at the edges of the Fourier transform window drastically alter the amplitude of the oscillations in the time domain, as shown in Fig. 5 ▸(*b*) and Figs. 2 ▸ and 3 ▸. In this context, it is suspected that incomplete knowledge of the detector efficiency, *e.g.* ageing detectors at high neutron energies, may be complicating the comparison.

The remaining mismatch in the high frequency range, *f*_M_ > 1000 Hz, is mainly attributed to the ToF data, which are basically a convolution of the broadening stemming from the sample and the instrument’s resolution function (elastic and inelastic). In ToF measurements, the resolution function *R*(2ϑ, *E*), which comprises contributions from the incident energy spread, initial pulse width, flight path length uncertainties, sample geometry, detector depth *etc.*, enters the experimentally measured *S*(2ϑ, *E*)_exp_ as a convolution: *S*(2ϑ, *E*)_exp_ = *R*(2ϑ, *E*) 
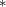
 *S*(2ϑ, *E*) (Zorn, 2012 ▸). Using the convolution theorem, the Fourier transform of this expression simplifies to *S*(2ϑ, *t*)_exp_ = *R*(2ϑ, *t*) *S*(2ϑ, *t*), making it in principle possible to deconvolute the measured data from resolution effects. However, to obtain a unique solution, all contributions to the instrumental resolution function – such as energy-dependent quantities like detector efficiency, polarization analysis and transmission coefficients – must be considered, which, unfortunately, is not feasible.

Fig. 5 ▸(*c*) compares the MIEZE transformation of the FOCUS and TOFTOF data, which were acquired utilizing two different sample containers: a flat Hellma macro-cell with an optical path length of 1 mm was used on FOCUS (and RESEDA), whereas a hollow cylinder with an optical path length of 0.15 mm was used on TOFTOF. The difference between the two data sets is significant. The solid brown line, showing the MIEZE transform of the data measured on TOFTOF, shows a plateau at much higher contrast values than the green curve showing the MIEZE transform of the data measured on FOCUS. A likely explanation is the difference in path length between the two samples, which leads to varying contributions from multiple scattering.

To circumvent the challenges of transforming ToF data into time/frequency space, an inverse Fourier transformation of the RESEDA data based on Bayesian analysis was attempted. This approach employed various regularization techniques that have been successfully used in the analysis of small-angle neutron scattering data (Bender *et al.*, 2017 ▸). This method profits from the fact that, for the MIEZE technique, the instrumental resolution can easily be disentangled from the signal contributions of the sample by normalizing it to the data of a purely elastic scatterer. However, this approach failed for energies beyond the SE approximation for several reasons. Firstly, for an accurate comparison, the transformed MIEZE data would need to be convoluted with the ToF resolution function. While a numerical approximation exists for large energies, the experimentally determined resolution function is mainly defined for small *E*. Secondly, the low data point density of the MIEZE measurement results in numerical artefacts that can only be addressed through strict regularization [see *e.g.* Bender *et al.* (2017 ▸)]. Thirdly, the reduced sensitivity of MIEZE at high energies makes the determination of a meaningful solution at high energies very challenging. Nevertheless, we acknowledge the capabilities of the analysis using an inverse Fourier transform and aim to apply it to an appropriately designed experiment in the future.

### 4.2. Indirect approach: parallel fit of model functions

A common approach to assess neutron scattering data is a standard *forward analysis*, meaning that an idealized model function is assumed and compared with the experimental data. This approach allows the simultaneous refinement of multiple data sets, such as those from different X-ray and neutron scattering techniques. In particular, it allows the fitting of data obtained from both MIEZE and ToF spectroscopy using a single model function. As a prerequisite, the resolution functions of each scattering technique must be properly incorporated. For ToF measurements, the elastic linewidth can be determined using a vanadium reference sample, while the resolution at finite energy transfers can only be estimated by means of analytical models or simulation. For TOFTOF this has been explicitly studied by Unruh *et al.* (2007 ▸) and Gaspar (2007 ▸). We will therefore follow this approach to compare the RESEDA data with the TOFTOF data. The main contributions to the uncertainty in the energy transfer were found to be the opening angles of the pulsing and monochromating choppers, the chopper rotation speeds, and the detector tubes. These last introduced uncertainties in terms of the detector’s dead time, differences in flight time due to the geometry and the wavelength-dependent detection efficiency of the tubes. The dependence on scattering angle was not included in the resolution function and was neglected in this treatment.

For MIEZE the situation is reversed: as mentioned in Section 3, the instrumental resolution is inherently included in the data reduction. Therefore, before transforming into the time/frequency domain, the model function only needs to be corrected for detector efficiency and detailed balance in the energy domain. The starting point for the parallel fit is a model consisting of analytical functions describing *S*(2ϑ, *E*), since *Q* cannot be clearly defined for large energy transfers in MIEZE. As the heuristic model a sum of Lorentzian distributions, 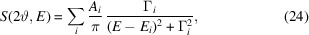
is employed. Here 2Γ_*i*_ is the linewidth (FWHM), *E*_*i*_ is either 0 (quasielastic contribution) or a finite energy transfer (inelastic contribution), and *A*_*i*_ is the contribution of the excitation to the total dynamic structure factor, normalized to unity (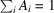).

**Figure 6:**
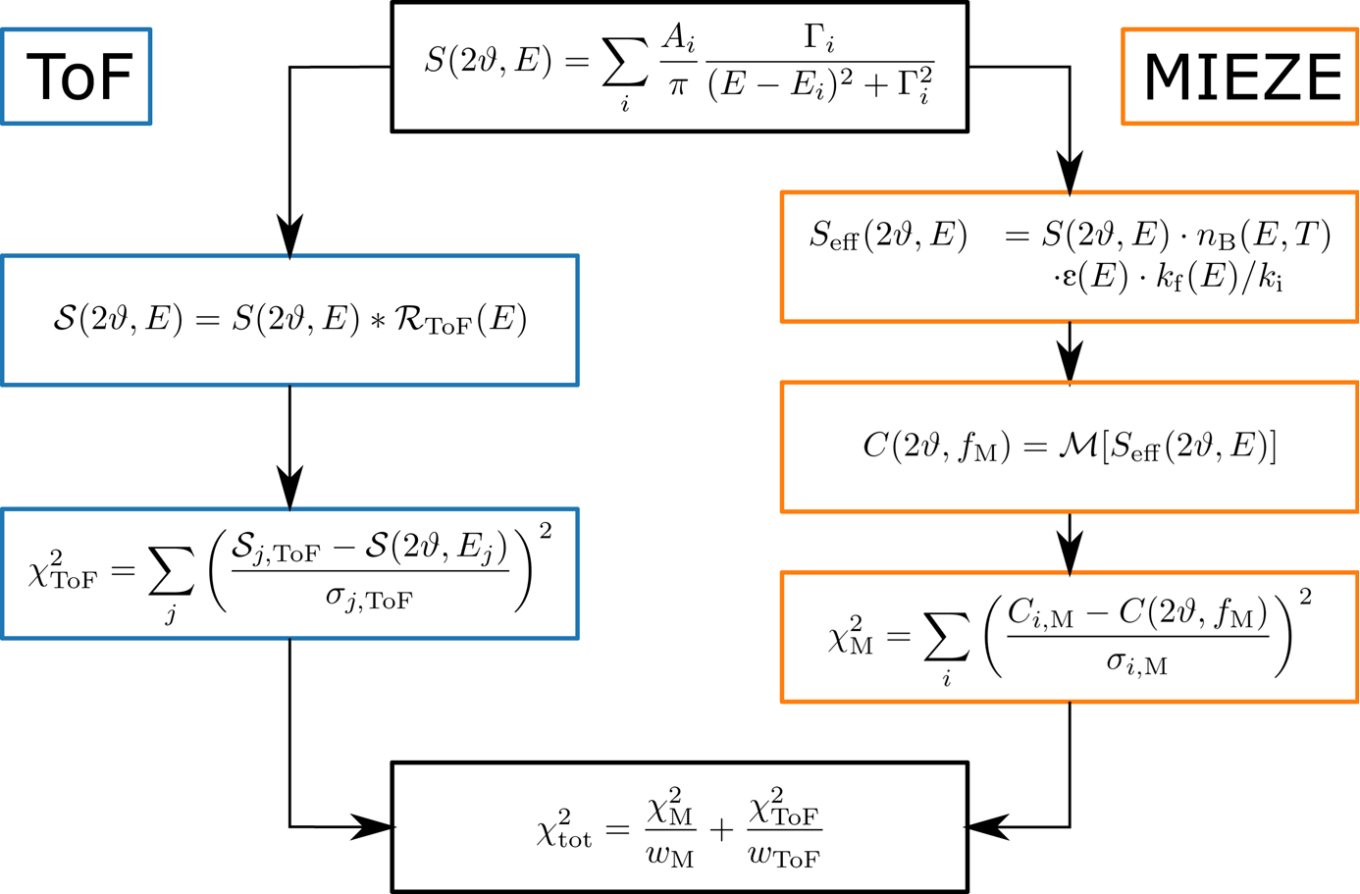
Flow chart of fitting combined data sets. The ToF procedure is depicted using blue boxes and the MIEZE procedure is highlighted using orange ones.

The treatment follows two pathways, one for each scattering technique, as depicted in Fig. 6 ▸. The blue and orange boxes represent the respective approaches, both originating from the heuristic model shown in the black box.

Starting with the blue path, the convolution of the heuristic model with the TOFTOF resolution function, 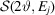, is given by 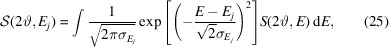
where 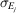 is the standard deviation expressing the instrumental uncertainty evaluated at the energy transfer *E*_*j*_. 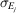 was calculated using the framework described by Gaspar (2007 ▸). Using this expression, the least-squares function for the TOFTOF data 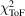 was computed (Fig. 6 ▸). For the sake of comparison, both the data set and the convolution models were normalized to unit area such that 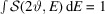.

Now following the orange path, the analysis of the MIEZE data required the transformation of the baseline model from the energy domain into the time domain. Especially in the case of large energy transfers, this required the computation of the contrast using equation (21). To comply with the theoretical framework laid out above for the baseline model, the detailed balance factor 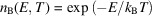 (where *k*_B_ is the Bolztmann constant), the detector efficiency ɛ(*E*) and the correction factor *k*_f_(*E*)/*k*_i_ were taken into account in the dynamic structure factor. This is marked in the upper orange box on the right-hand side of Fig. 6 ▸. Subsequently, the MIEZE transformation, denoted 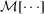, was calculated using the effective structure factor *S*_eff_(2ϑ, *E*) in equation (21). Then the least-squares value of the MIEZE data, 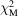, is computed.

**Figure 7:**
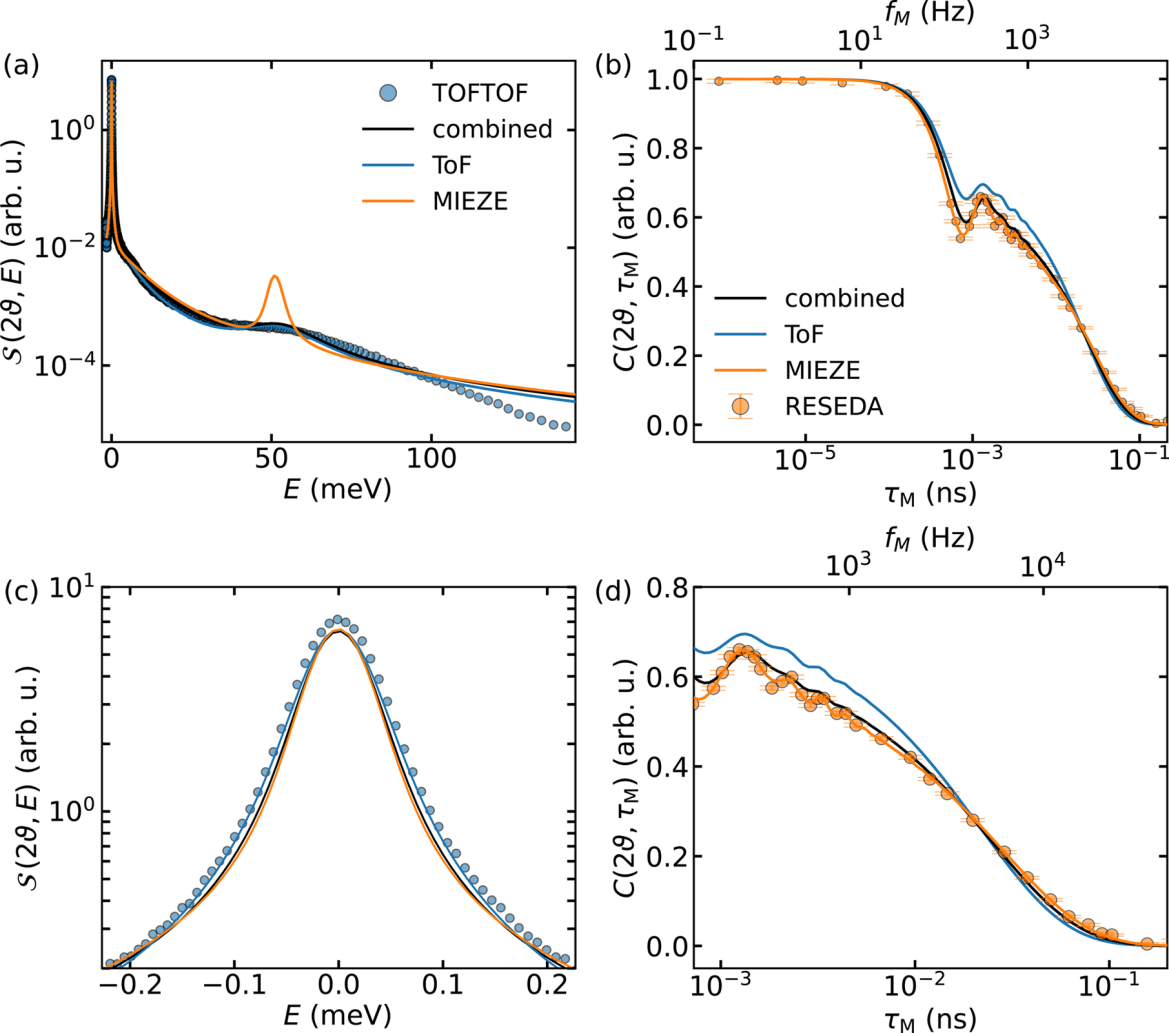
Results of fitting the 3Q1I model. The points in blue and orange visualize measurements on the TOFTOF and RESEDA spectrometers, respectively. Where error bars are not shown, they are significantly smaller than the size of the markers. The solid lines represent the results of the fit analyses. The orange line represents the fit of the RESEDA data, convoluted with the instrument resolution for comparison with the TOFTOF data. Similarly, the blue line represents the fit of the TOFTOF data, transformed for comparison with the RESEDA data. The fit of the MIEZE data is insensitive to the inelastic scattering contribution, which is why the amplitude is overestimated to improve the fit at low energy transfers. The black line shows the result of the combined fitting. Panels (*a*) and (*b*) show the entire dynamic range of both measurements, whereas panels (*c*) and (*d*) give more detailed views of the parameter space where the SE approximation is valid.

Three variations of the heuristic Lorentzian model, consisting of two quasielastic + one inelastic peak (2Q1I), three quasielastic peaks (3Q) and three quasielastic + one inelastic peaks (3Q1I), have been fitted to the combined data set. As this last has shown the best agreement with the data, we will limit our discussions to model 3Q1I. The result of this procedure is presented in Fig. 7 ▸. While the combined fit captures the main features of both data sets, the agreement between the data and the fit could be improved. The discrepancy most likely arises from the different sample thicknesses used at TOFTOF and RESEDA, leading to varying contributions from multiple scattering. When comparing the independent fits of MIEZE and ToF data in the energy domain, reasonably good agreement between the two fits is achieved, with the exception of the sharp peak at *E* ≃ 50 meV. Unfortunately, the MIEZE data are not very sensitive to the large energy transfer of the inelastic contribution observed in the contrast. The inelastic contribution ought to appear in the three or four MIEZE data points around the contrast drop at 10^−4^ ns. However, since its relative contribution to the total dynamic structure factor is ∼2%, the corresponding variation in the contrast is of the order of the statistical uncertainty of the experiment. As a result, an unconstrained fit of the 3Q1I model to the RESEDA data converges to a model comprising four superimposed quasielastic distributions. For this reason, we analysed the RESEDA data with a model where *E* ≃ 50.1 meV was fixed for the inelastic contribution. This value corresponds to the result of the ToF fit. A higher data point density around the drop in contrast could improve the MIEZE fit.

Summing 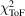 and 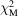 yields the total least-squares value 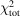, which should be minimized by the optimal model through variation of the free parameters *A*_*i*_, *E*_*i*_ and Γ_*i*_ of each Lorentzian peak. However, adding up 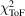 and 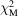 is not straightforward. Due to the comparatively large number of data points and the smaller relative uncertainty, 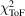 dominates the combined least-squares function. While this has far reaching consequences, especially in the context of the estimated uncertainties, we opted to weight χ^2^ by 1/*N*, with *N* being the number of data points.

The model parameters were optimized numerically using the differential evolution algorithm (Storn & Price, 1997 ▸) implemented in the *SciPy* (Virtanen *et al.*, 2020 ▸) library and the Python wrapper package *iminuit*, which makes the *MIGRAD* algorithm of CERN’s *ROOT* data analysis framework available (Dembinski *et al.*, 2020 ▸; Antcheva *et al.*, 2011 ▸). The uncertainties in the fitting parameters obtained were evaluated using an inverse Hessian matrix and population statistics acquired by differential evolution fitting, as described in Appendix *A*[App appa].

## 5. Further discussion

Several pitfalls that significantly affect the transformation and thus complicate direct comparisons between the methods have been identified and need careful consideration. These aspects have been demonstrated using the extreme examples in Section 2.3 and will be discussed in more detail here.

### 5.1. Sample geometry

The sample geometry (Zorn, 2012 ▸) – or more specifically, the sample thickness in the context of this study – has been identified as a significant challenge when directly comparing ToF and MIEZE data. This is shown in Fig. 5 ▸(*c*), where the MIEZE transformations of the FOCUS and TOFTOF data, which were acquired utilizing two different sample containers, are compared with the RESEDA data. A likely explanation is the difference in path length between the two samples, which leads to varying contributions from multiple scattering. This was shown for example by Busch & Unruh (2011 ▸), who investigated the effects of sample thickness on the (intermediate) scattering function of H_2_O. The authors found that, in thicker layers, the increased contribution to multiple scattering broadens the experimentally determined linewidth in *S*(*Q*, *E*), corresponding to a shorter relaxation time in *I*(*Q*, τ). A detailed analysis of the influence of different sample shapes on ToF data has been performed by Zorn *et al.* (2012 ▸) but is beyond the scope of this article.

### 5.2. Background signal

In addition to the geometry of the sample, effects such as the energy resolution of the ToF spectrometer, the character of the background and control over the latter during the data reduction process influence the transformation. Dark counts in the detector electronics, spurious scattering from *e.g.* sample environment components or air, and cross talk due to coarse collimation are certainly difficult to characterize, often leaving a background that cannot be neglected. As a final significant influence, accurate knowledge of the neutron detection efficiency, and of the spin-flip ratio of the analyser and its dependence on the neutron energy, is required when analysing data beyond the SE approximation.

### 5.3. Outlook

**Figure 8:**
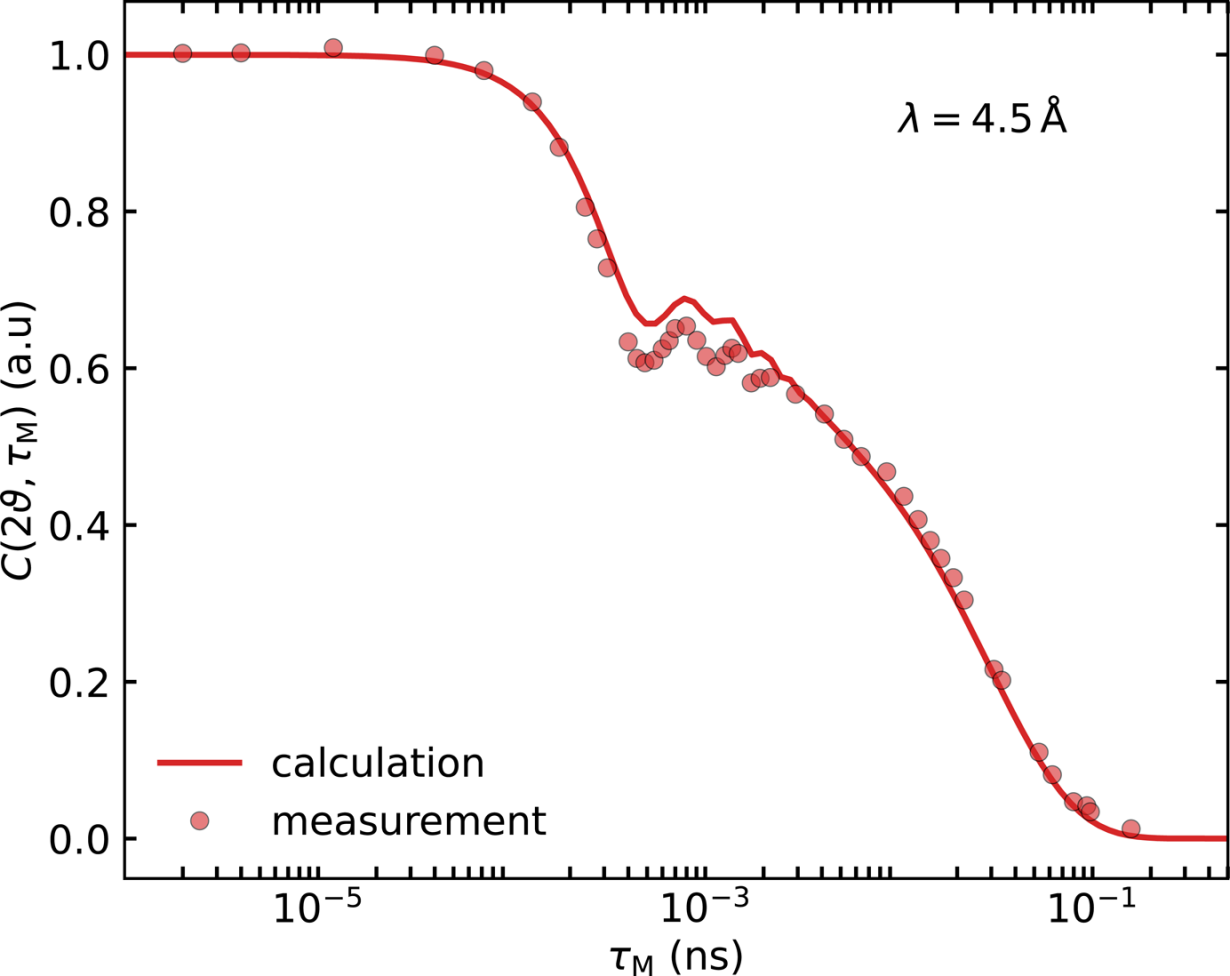
Comparison of the predicted contrast calculated from the optimized 3Q1I model (Section 4.2) for data at λ_i_ = 4.5 Å recorded on RESEDA. The data were collected at an angle 2ϑ such that the momentum transfer *Q* is the same in the SE approximation. The predicted curves account for the increased integration area and the adjustments in the MIEZE phase calculation.

The transformation presented here can be readily applied to various approaches for analysing and designing experiments. It allows *e.g.* MD simulation outputs to be converted into expected MIEZE spectra, facilitating direct comparison with experimental data and allowing one to distinguish between competing models. Using model 3Q1I (Section 4.2) as an example, this approach is demonstrated as a means of guiding experiment design, ensuring that appropriate timescales and data point distributions are chosen for meaningful validation of simulation results. Fig. 8 ▸ presents the results of transforming the model used in Fig. 7 ▸, which was fitted to the data acquired at λ_i_ = 6.0 Å while accounting for the different λ_i_ = 4.5 Å setting. The prediction (red line) without any adjustment of model parameters is in good agreement with the measured data using neutrons with λ_i_ = 4.5 Å, except for the precise vertical position of the oscillations. The oscillations, which are in this case a consequence of the MIEZE method, are a source of instability in the fitting process and possibly lead to increased uncertainty in the model parameters.

**Figure 9:**
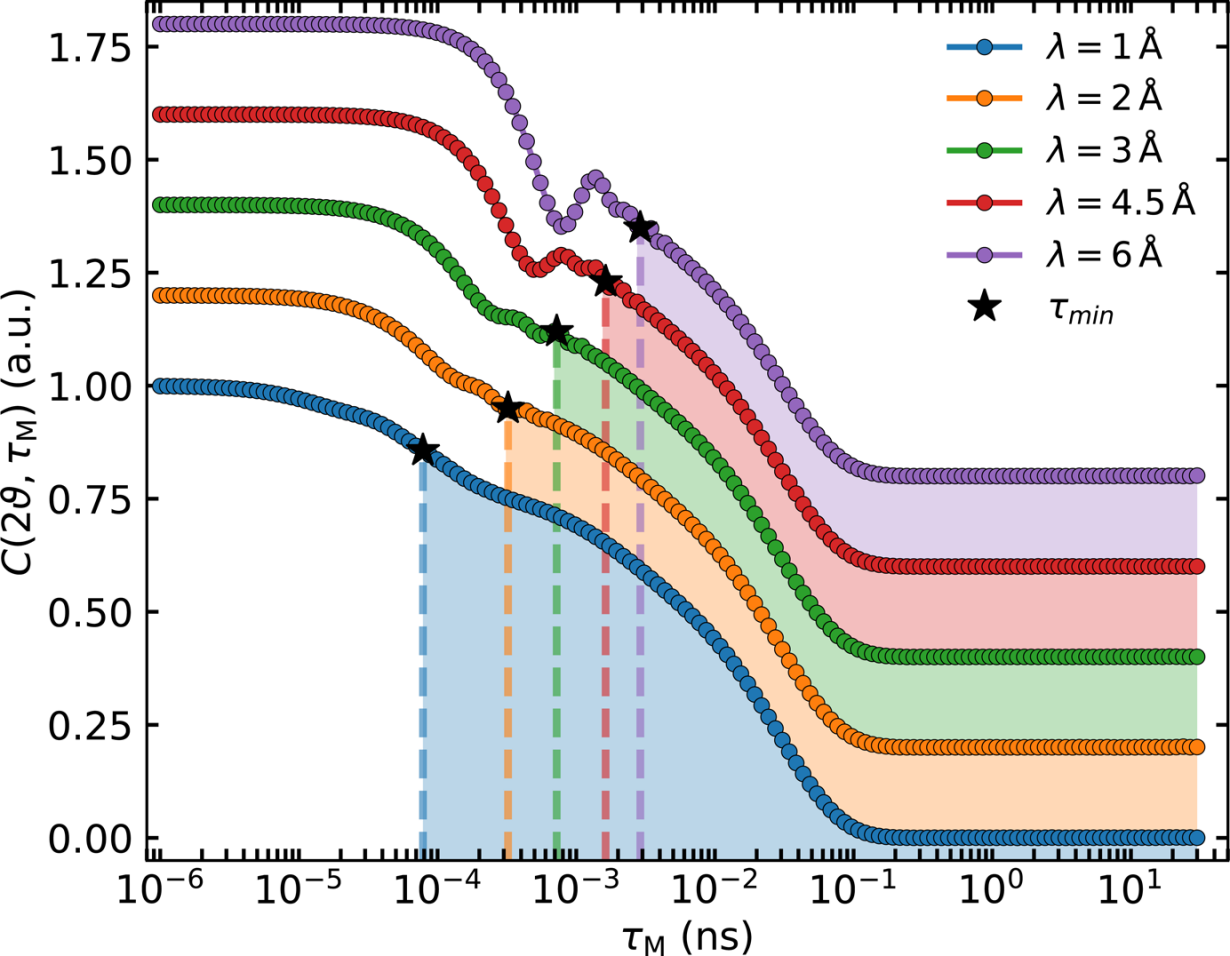
Prediction for typical data measured on RESEDA for different initial wavelengths λ_i_. The purple curve corresponds to the results of fitting the TOFTOF data with model 3Q1I. This result was then used to predict MIEZE data for different wavelengths, similar to Fig. 8. The star labelled τ_min_ indicates the smallest Fourier time, below which the SE approximation becomes invalid, at the corresponding wavelength λ_i_.

However, as demonstrated in Section 2.3, a higher kinetic energy of incoming neutrons suppresses these unwanted features, increasing the reliability of the data analysis. Fig. 9 ▸ visualizes this effect. The different curves correspond to a transformation of the same model function as before with decreasing wavelength λ_i_. The star marks the MIEZE time τ_min_ = ℏ/(0.1*E*_i_) below which the SE approximation is no longer valid. This capability will become available when implementing the proposed instrument upgrade to TIGER (Jochum *et al.*, 2022 ▸) at RESEDA.

The parallel fitting procedure will also be highly beneficial for a MIEZE instrument at a pulsed neutron source. The broad wavelength range readily available at such a source will enable the parallel fitting of multiple wavelengths, leading to a more precise determination of the model parameters. As shown in Fig. 9 ▸, varying wavelengths allow probing of the region of *I*(*Q*, τ) that is most sensitive to the energy cutoff, facilitating the study of inelastic signals with exceptional energy resolution.

## 6. Conclusions

In conclusion, two methods for comparing ToF and NSE/MIEZE data have been introduced. The first approach is based on a direct transformation, enabling a straightforward comparison between excitation spectra measured in a ToF instrument and their counterparts observed by a MIEZE spectrometer in the time domain. Several factors that become important when considering large energy transfers relative to the kinetic energy of the neutron, such as detector efficiencies and energy conservation (*k*_f_/*k*_i_), are taken into account. This framework has been successfully applied to data acquired on an ultra-pure water sample at room temperature using the ToF spectrometers FOCUS and TOFTOF, and the results have been compared with data measured on the MIEZE instrument RESEDA. Under identical sample conditions, convincing agreement is observed between the transformed ToF data and the MIEZE data.

In addition to enabling direct comparisons between data sets from different instruments, this approach also allows data from numerical calculations to be transformed to predict MIEZE measurement outcomes for given experimental conditions. This capability will further support the efficient planning of future experiments.

The second approach employs a combined fit of model functions to describe both data sets. This method provides an opportunity to test a model against data measured in both the energy and time domains while accounting for detector efficiency, instrumental resolution and detailed balance. A persistent challenge is the presence of measurement artefacts that cannot be determined empirically or through numerical calculations, such as detector dark counts and other energy-independent backgrounds.

As a final conclusion, we emphasize the complementarity of MIEZE and ToF and the importance of ensuring quantitative consistency between data collected with these techniques. While MIEZE offers significantly higher energy resolution, ToF provides broader coverage of *Q*–*E* space in a shorter measurement time. MIEZE is ideally suited for studying dynamic processes at small scattering angles, whereas ToF allows for a clearer investigation of dynamics at large energy transfers which would pose a challenge to the MIEZE technique alone.

## Appendix A Uncertainty analysis

Using only a single data set, one can employ the common uncertainty estimation in terms of a quadratic approximation of the least-squares cost function around the minimum. For the least-squares analysis, the 1σ uncertainty estimate and covariance of a fit parameter *a*_*i*_ are given by the contour in parameter space where . This contour can be traced by mapping out the least-squares cost function around the minimum, which becomes exceedingly time consuming in cases of a high-dimensional space of fitting parameters. More efficient is the calculation of a quadratic approximation of the least-squares function using the Hessian matrix **H** adopted in gradient descent or related minimization algorithms. The standard uncertainty of a fit parameter *a*_*i*_ is then calculated as . Ideally, the fit of an appropriate model function to a set of data points and their standard deviation (*y*_*i*_, σ_*i*_) should result in a goodness-of-fit value *g*_fit_ ≃ 1, which is calculated as

similar to the reduced χ^2^ value. However, the σ_*i*_ values do not necessarily capture all uncertainty associated with the value *y*_*i*_, leading to a *g*_fit_ significantly larger than 1. *n* is the number of data points and *m* is the number of fitting parameters included in the model function *f*. In a first attempt to account for this fact, *g*_fit_ can be used as a scaling factor between the weights in the least-squares function *w*_*i*_ and the σ_*i*_ associated with each data point: . Then, σ_*i*_ represent the relative uncertainties between the data points, but not the ‘total uncertainty’. As a result, the uncertainties in the fitting parameters need to be re-scaled to  as well (Strutz, 2011 ▸).

The method of estimating the reliability of the fitting parameters assumes certain statistics of the data points used in the computation of the χ^2^ value. In case of the combined model with , the unnatural emphasis of the MIEZE data skews the results compared with a standard least-squares function. Thus, besides the uncertainty estimates obtained from the calculations described above, we used the intermediate results of the differential evolution algorithm to study the distribution of fitting parameters, which yielded . The populations of vectors in parameter space, which are drawn by the differential evolution algorithm, were produced via the *best1bin* strategy (Storn & Price, 1997 ▸). The uncertainty in each optimal parameter is then estimated by calculating the standard deviation of the marginalized distribution with respect to the optimal value. For a 2% deviation from the minimal χ^2^, between 200 and 400 parameter vectors are used for this analysis.

## References

[bb1] Andersen, K. (1996). *Nucl. Instrum. Methods Phys. Res. A*, **371**, 472–479.

[bb2] Antcheva, I., Ballintijn, M., Bellenot, B., Biskup, M., Brun, R., Buncic, N., Canal, P., Casadei, D., Couet, O., Fine, V., Franco, L., Ganis, G., Gheata, A., Maline, D. G., Goto, M., Iwaszkiewicz, J., Kreshuk, A., Segura, D. M., Maunder, R., Moneta, L., Naumann, A., Offermann, E., Onuchin, V., Panacek, S., Rademakers, F., Russo, P. & Tadel, M. (2011). *Comput. Phys. Commun.* **182**, 1384–1385.

[bb3] Arnold, O., Bilheux, J., Borreguero, J., Buts, A., Campbell, S., Chapon, L., Doucet, M., Draper, N., Ferraz Leal, R., Gigg, M., Lynch, V., Markvardsen, A., Mikkelson, D., Mikkelson, R., Miller, R., Palmen, K., Parker, P., Passos, G., Perring, T., Peterson, P., Ren, S., Reuter, M., Savici, A., Taylor, J., Taylor, R., Tolchenov, R., Zhou, W. & Zikovsky, J. (2014). *Nucl. Instrum. Methods Phys. Res. A*, **764**, 156–166.

[bb4] Azuah, R. T., Kneller, L. R., Qiu, Y., Tregenna-Piggott, P. L., Brown, C. M., Copley, J. R. & Dimeo, R. M. (2009). *J. Res. Natl Inst. Stand. Technol.* **114**, 341–358. 10.6028/jres.114.025 PMC4646530 27504233

[bb5] Bee, M. (2025). *Quasielastic neutron scattering.* Adam Hilger.

[bb6] Bender, P., Bogart, L., Posth, O., Szczerba, W., Rogers, S. E., Castro, A., Nilsson, L., Zeng, L., Sugunan, A., Sommertune, J., Fornara, A., González-Alonso, D., Fernández Barquín, L. & Johansson, C. (2017). *Sci. Rep.* **7**, 45990. 10.1038/srep45990 PMC5387715 28397851

[bb7] Bewley, R., Taylor, J. & Bennington, S. (2011). *Nucl. Instrum. Methods Phys. Res. A*, **637**, 128–134.

[bb8] Biehl, R. & Richter, D. (2014). *J. Phys. Condens. Matter*, **26**, 503103. 10.1088/0953-8984/26/50/503103 25419898

[bb9] Bu, Z., Biehl, R., Monkenbusch, M., Richter, D. & Callaway, D. J. E. (2005). *Proc. Natl Acad. Sci. USA*, **102**, 17646–17651. 10.1073/pnas.0503388102 PMC1345721 16306270

[bb10] Busch, S. & Unruh, T. (2011). *J. Phys. Condens. Matter*, **23**, 254205.

[bb11] Dembinski, H., Ongmongkolkul, P., Deil, C., Schreiner, H., Feickert, M., Burr, C., Watson, J., Rost, F., Pearce, A., Geiger, L., Abdelmotteleb, A., Desai, A., Wiedemann, B. M., Gohlke, C., Sanders, J., Drotleff, J., Eschle, J., Neste, L., Gorelli, M. E., Baak, M., Eliachevitch, M. & Zapata, O. (2020). *scikit-hep/iminuit*, https://doi.org/10.5281/zenodo.3949207.

[bb12] Demmel, F., McPhail, D., French, C., Maxwell, D., Harrison, S., Boxall, J., Rhodes, N., Mukhopadhyay, S., Silverwood, I., Sakai, V. G. & Fernandez-Alonso, F. (2018). *J. Phys. Conf. Ser.* **1021**, 012027.

[bb13] Farago, B., Falus, P., Hoffmann, I., Gradzielski, M., Thomas, F. & Gomez, C. (2015). *Neutron News*, **26**(3), 15–17.

[bb14] Fouquet, P., Ehlers, G., Farago, B., Pappas, C. & Mezei, F. (2007). *J. Neutron Res.* **15**, 39–47.

[bb15] Franz, C., Säubert, S., Wendl, A., Haslbeck, F. X., Soltwedel, O., Jochum, J. K., Spitz, L., Kindervater, J., Bauer, A., Böni, P. & Pfleiderer, C. (2019*a*). *J. Phys. Soc. Jpn*, **88**, 081002.

[bb16] Franz, C. & Schröder, T. (2015). *J. Large-Scale Res. Facil.* **1**, A14.

[bb17] Franz, C., Soltwedel, O., Fuchs, C., Säubert, S., Haslbeck, F., Wendl, A., Jochum, J. K., Böni, P. & Pfleiderer, C. (2019*b*). *Nucl. Instrum. Methods Phys. Res. A*, **939**, 22–29.

[bb18] Franz, C., Soltwedel, O., Säubert, S., Wendl, A., Gottwald, W., Haslbeck, F., Spitz, L. & Pfleiderer, C. (2019*c*). *J. Phys. Conf. Ser.* **1316**, 012005.

[bb19] Frick, B., Mamontov, E., Eijck, L. & Seydel, T. (2010). *Z. Phys. Chem.* **224**, 33–60.

[bb20] Gähler, R., Golub, R. & Keller, T. (1992). *Physica B*, **180–181**, 899–902.

[bb21] Gardner, J. S., Ehlers, G., Faraone, A. & García Sakai, V. (2020). *Nat. Rev. Phys.* **2**, 103–116.

[bb22] Gaspar, A. M. (2007). *arXiv*, 0710.5319.

[bb23] Golub, R. & Gähler, R. (1987). *Phys. Lett. A*, **123**, 43–48.

[bb25] Golub, R., Gähler, R. & Keller, T. (1994). *Am. J. Phys.* **62**, 779–788.

[bb28] Häussler, W. & Schmidt, U. (2005). *Phys. Chem. Chem. Phys.* **7**, 1245–1249. 10.1039/b419281h 19791340

[bb26] Holderer, O. & Ivanova, O. (2015). *J. Large-Scale Res. Facil.* **1**, A11.

[bb27] Hopfenmüller, B., Zorn, R., Holderer, O., Ivanova, O., Lehnert, W., Lüke, W., Ehlers, G., Jalarvo, N., Schneider, G. J., Monkenbusch, M. & Richter, D. (2018). *J. Chem. Phys.* **148**, 204906. 10.1063/1.5018717 29865825

[bb29] Inoue, R., Biehl, R., Rosenkranz, T., Fitter, J., Monkenbusch, M., Radulescu, A., Farago, B. & Richter, D. (2010). *Biophys. J.* **99**, 2309–2317. 10.1016/j.bpj.2010.08.017 PMC3042550 20923666

[bb30] Janßen, S., Mesot, J., Holitzner, L., Furrer, A. & Hempelmann, R. (1997). *Physica B*, **234–236**, 1174–1176.

[bb31] Jochum, J. K., Franz, C., Keller, T. & Pfleiderer, C. (2022). *J. Appl. Cryst.* **55**, 1424–1431. 10.1107/S1600576722009505 PMC9721327 36570654

[bb32] Jochum, J. K., Wendl, A., Keller, T. & Franz, C. (2019). *Meas. Sci. Technol.* **31**, 035902.

[bb33] Kanaya, T., Kaji, K., Kitamaru, R., Higgins, J. S. & Farago, B. (1989). *Macromolecules*, **22**, 1356–1359.

[bb34] Keller, T., Golub, R. & Gähler, R. (2002). *Scattering*, edited by R. Pike & P. Sabatier, p. 1264. Cambridge: Academic Press.

[bb35] Keller, T., Trepka, H., Habicht, K. & Keimer, B. (2022). *Phys. Status Solidi B*, **259**, 2100164.

[bb36] Köhli, M., Klein, M., Allmendinger, F., Perrevoort, A.-K., Schröder, T., Martin, N., Schmidt, C. J. & Schmidt, U. (2016). *J. Phys. Conf. Ser.* **746**, 012003.

[bb37] Kyrey, T., Witte, J., Feoktystov, A., Pipich, V., Wu, B., Pasini, S., Radulescu, A., Witt, M. U., Kruteva, M., von Klitzing, R., Wellert, S. & Holderer, O. (2019). *Soft Matter*, **15**, 6536–6546. 10.1039/c9sm01161g 31355828

[bb38] Le, M. D., Guidi, T., Bewley, R. I., Stewart, J. R., Schooneveld, E. M., Raspino, D., Pooley, D. E., Boxall, J., Gascoyne, K. F., Rhodes, N. J., Moorby, S. R., Templeman, D. J., Afford, L. C., Waller, S. P., Zacek, D. & Shaw, R. C. R. (2023). *Nucl. Instrum. Methods Phys. Res. A*, **1056**, 168646.

[bb39] Mesot, J., Janssen, S., Holitzner, L. & Hempelmann, R. (1996). *J. Neutron Res.* **3**, 293–310.

[bb40] Mezei, F. (1972). *Z. Phys. A*, **255**, 146–160.

[bb41] Mezei, F. (1980). *Neutron spin echo*. Lecture notes in physics, Vol. 128. Berlin, Heidelberg: Springer.

[bb42] Mezei, F., Drabkin, G. & Ioffe, A. (2001). *Physica B*, **297**, 9–13.

[bb43] Mezei, F., Pappas, C. & Gutberlet, T. (2003). *Neutron spin echo spectroscopy: basics, trends and applications*, Lecture notes in physics, Vol. 601. Berlin: Springer.

[bb44] Mihailescu, M., Monkenbusch, M., Endo, H., Allgaier, J., Gompper, G., Stellbrink, J., Richter, D., Jakobs, B., Sottmann, T. & Farago, B. (2001). *J. Chem. Phys.* **115**, 9563–9577.

[bb45] Monkenbusch, M. (1990). *Nucl. Instrum. Methods Phys. Res. A*, **287**, 465–475.

[bb46] Nilsen, G. J., Košata, J., Devonport, M., Galsworthy, P., Bewley, R. I., Voneshen, D. J., Dalgliesh, R. & Stewart, J. R. (2017). *J. Phys. Conf. Ser.* **862**, 012019.

[bb47] Nylander, T., Soltwedel, O., Ganeva, M., Hirst, C., Holdaway, J., Arteta, M. Y., Wadsäter, M., Barauskas, J., Frielinghaus, H. & Holderer, O. (2017). *J. Phys. Chem. B*, **121**, 2705–2711. 10.1021/acs.jpcb.6b11038 28266854

[bb48] Ollivier, J., Casalta, H., Schober, H., Cook, J., Malbert, P., Locatelli, M., Gomez, C., Jenkins, S., Sutton, I. & Thomas, M. (2002). *Appl. Phys. Mater. Sci. Process.* **74**, s305–s307.

[bb49] Ollivier, J. & Mutka, H. (2011). *J. Phys. Soc. Jpn*, **80**(Suppl. B), SB003.

[bb50] Pappas, C., Kali, G., Böni, P., Kischnik, R., Mertens, L., Granz, P. & Mezei, F. (2000). *Physica B*, **276–278**, 162–163.

[bb51] Pasini, S., Holderer, O., Kozielewski, T., Richter, D. & Monkenbusch, M. (2019). *Rev. Sci. Instrum.* **90**, 043107. 10.1063/1.5084303 31043036

[bb52] Richter, D., Arbe, A., Colmenero, J., Monkenbusch, M., Farago, B. & Faust, R. (1998). *Macromolecules*, **31**, 1133–1143. 10.1021/ma9717810 9680431

[bb53] Salatto, D., Carrillo, J. Y., Endoh, M. K., Taniguchi, T., Yavitt, B. M., Masui, T., Kishimoto, H., Tyagi, M., Ribbe, A. E., Garcia Sakai, V., Kruteva, M., Sumpter, B. G., Farago, B., Richter, D., Nagao, M. & Koga, T. (2021). *Macromolecules*, **54**, 11032–11046.

[bb54] Schleger, P., Farago, B., Lartigue, C., Kollmar, A. & Richter, D. (1998). *Phys. Rev. Lett.* **81**, 124–127.

[bb55] Schober, A., Wendl, A., Haslbeck, F. X., Jochum, J. K., Spitz, L. & Franz, C. (2019). *J. Phys. Commun.* **3**, 103001.

[bb56] Storn, R. & Price, K. (1997). *J. Glob. Optim.* **11**, 341–359.

[bb57] Strutz, T. (2011). *Data fitting and uncertainty: a practical introduction to weighted least squares and beyond.* Heidelberg: Springer.

[bb58] Unke, O. T., Chmiela, S., Sauceda, H. E., Gastegger, M., Poltavsky, I., Schütt, K. T., Tkatchenko, A. & Müller, K.-R. (2021). *Chem. Rev.* **121**, 10142–10186. 10.1021/acs.chemrev.0c01111 PMC8391964 33705118

[bb59] Unruh, T., Neuhaus, J. & Petry, W. (2007). *Nucl. Instrum. Methods Phys. Res. A*, **580**, 1414–1422.

[bb60] Virtanen, P., Gommers, R., Oliphant, T. E., Haberland, M., Reddy, T., Cournapeau, D., Burovski, E., Peterson, P., Weckesser, W., Bright, J., van der Walt, S. J., Brett, M., Wilson, J., Millman, K. J., Mayorov, N., Nelson, A. R. J., Jones, E., Kern, R., Larson, E., Carey, C. J., Polat, İ., Feng, Y., Moore, E. W., VanderPlas, J., Laxalde, D., Perktold, J., Cimrman, R., Henriksen, I., Quintero, E. A., Harris, C. R., Archibald, A. M., Ribeiro, A. H., Pedregosa, F., van Mulbregt, P., Vijaykumar, A., Bardelli, A. P., Rothberg, A., Hilboll, A., Kloeckner, A., Scopatz, A., Lee, A., Rokem, A., Woods, C. N., Fulton, C., Masson, C., Häggström, C., Fitzgerald, C., Nicholson, D. A., Hagen, D. R., Pasechnik, D. V., Olivetti, E., Martin, E., Wieser, E., Silva, F., Lenders, F., Wilhelm, F., Young, G., Price, G. A., Ingold, G., Allen, G. E., Lee, G. R., Audren, H., Probst, I., Dietrich, J. P., Silterra, J., Webber, J. T., Slavič, J., Nothman, J., Buchner, J., Kulick, J., Schönberger, J. L., de Miranda Cardoso, J. V., Reimer, J., Harrington, J., Rodríguez, J. L. C., Nunez-Iglesias, J., Kuczynski, J., Tritz, K., Thoma, M., Newville, M., Kümmerer, M., Bolingbroke, M., Tartre, M., Pak, M., Smith, N. J., Nowaczyk, N., Shebanov, N., Pavlyk, O., Brodtkorb, P. A., Lee, P., McGibbon, R. T., Feldbauer, R., Lewis, S., Tygier, S., Sievert, S., Vigna, S., Peterson, S., More, S., Pudlik, T., Oshima, T., Pingel, T. J., Robitaille, T. P., Spura, T., Jones, T. R., Cera, T., Leslie, T., Zito, T., Krauss, T., Upadhyay, U., Halchenko, Y. O. & Vázquez-Baeza, Y. (2020). *Nat. Methods*, **17**, 261–272.

[bb61] Walter, W., Boffo, C., Borlein, M., Kozielewski, T., Monkenbusch, M., Ohl, M., Paul, A., Schrauth, B., Sikler, G. & Tiemann, C. (2009). *IEEE Trans. Appl. Supercond.* **19**, 1320–1323.

[bb62] Winn, B., Filges, U., Garlea, V. O., Graves-Brook, M., Hagen, M., Jiang, C., Kenzelmann, M., Passell, L., Shapiro, S. M., Tong, X. & Zaliznyak, I. (2015). *EPJ Web Conf.* **83**, 03017.

[bb63] Wuttke, J., Petry, W., Coddens, G. & Fujara, F. (1995). *Phys. Rev. E*, **52**, 4026–4034. 10.1103/physreve.52.4026 9963875

[bb64] Zaliznyak, I., Ghosh, V., Shapiro, S. & Passell, L. (2005). *Physica B*, **356**, 150–155.

[bb65] Zolnierczuk, P. A., Holderer, O., Pasini, S., Kozielewski, T., Stingaciu, L. R. & Monkenbusch, M. (2019). *J. Appl. Cryst.* **52**, 1022–1034. 10.1107/S1600576719010847 PMC6782076 31636520

[bb66] Zorn, R. (2012). *Nucl. Instrum. Methods Phys. Res. A*, **674**, 85–91.

